# CaSWC4 regulates the immunity-thermotolerance tradeoff by recruiting CabZIP63/CaWRKY40 to target genes and activating chromatin in pepper

**DOI:** 10.1371/journal.pgen.1010023

**Published:** 2022-02-28

**Authors:** Weiwei Cai, Sheng Yang, Ruijie Wu, Yutong Zheng, Shicong He, Lei Shen, Deyi Guan, Shuilin He

**Affiliations:** 1 Key Laboratory of Applied Genetics of universities in Fujian Province, Fujian Agriculture and Forestry University, Fuzhou, Fujian, PR China; 2 National Education Ministry Key Laboratory of Plant Genetic Improvement and Comprehensive Utilization, Fujian Agriculture and Forestry University, Fuzhou, Fujian, PR China; 3 Agricultural College, Fujian Agriculture and Forestry University, Fuzhou, Fujian, PR China; 4 College of of Horticultural Sciences, Zhejiang Agriculture and Forestry University, Hangzhou, Zhejiang, PR China; Tsinghua University, CHINA

## Abstract

Pepper (*Capsicum annuum*) responds differently to high temperature stress (HTS) and *Ralstonia solanacearum* infection (RSI) but employs some shared transcription factors (TFs), such as CabZIP63 and CaWRKY40, in both cases. How the plant activates and balances these distinct responses, however, was unclear. Here, we show that the protein CaSWC4 interacts with CaRUVBL2 and CaTAF14b and they all act positively in pepper response to RSI and thermotolerance. CaSWC4 activates chromatin of immunity or thermotolerance related target genes of CaWRKY40 or CabZIP63 by promoting deposition of H2A.Z, H3K9ac and H4K5ac, simultaneously recruits CabZIP63 and CaWRKY40 through physical interaction and brings them to their targets (immunity- or thermotolerance-related genes) via binding AT-rich DNA element. The above process relies on the recruitment of CaRUVBL2 and TAF14 by CaSWC4 via physical interaction, which occurs at loci of immunity related target genes only when the plants are challenged with RSI, and at loci of thermotolerance related target genes only upon HTS. Collectively, our data suggest that CaSWC4 regulates rapid, accurate responses to both RSI and HTS by modulating chromatin of specific target genes opening and recruiting the TFs, CaRUVBL2 and CaTAF14b to the specific target genes, thereby helping achieve the balance between immunity and thermotolerance.

## Author summary

Transcription mediated by different transcription factors (TFs) and chromatin remodeling during plant stress response is currently one of the most intensive studied topics, but how transcription factors associate with chromatin remodeling modulators in coordinating responses of plant to multiple stresses remain poorly understood. Herein, using pepper response to RSI and to HTS as an example, we studied how CaWRKY40 and CabZIP63, the two TFs that positively regulate pepper immunity against RSI and termotolerance, associate with SWR1-c and other chromatin remodeling modulators in coordinating responses of plant immunity and thermotolerance. Our results indicate that CaSWC4, a component shared by SWR1- and NuA4 complex, activates the chromatin loci of the tested immunity and thermotolerance related genes targeted by CaWRKY40 and CabZIP63 through promoting the deposition of chromatin active markers including H2A.Z, H3H9ac and H4K5ac, and bring CaWRKY40 and CabZIP63 to the promoters of their target genes by physical interactions. In addition, through interaction between CaSWC4 and CaRUVBL2/CaTAF14b and their co-deposition to the target genes of CaWRKY40 and CabZIP63 in a stress dependent manner, immunity against RSI or thermotolerance is specifically and accurately activated by CaWRKY40/CabZIP63-CaSWC4 nodule. Our results provide new insight into the mechanism underlying plant immunity, thermotolerance and their balance.

## Introduction

Plants are frequently exposed to various biotic and abiotic stresses simultaneously or successively and must appropriately respond to these stresses in order to optimize their fitness. Thus, plants must allocate their limited resources to address the most life-threatening stresses via diverse mechanisms and tradeoffs. These tradeoffs are dynamically modulated according to changes in the lethality of various stresses [1, 2]; [3, 4]. However, the players in these tradeoffs and the underlying molecular details remain largely unknown.

High temperature stress (HTS) and pathogen attack frequently retard plant growth and development. Plants activate distinct defense responses to defend themselves against these stresses [5–9]. Plants employ pattern recognition receptors (PRRs) at the plasma membrane and intracellular R-proteins to perceive pathogens by recognizing pathogen-derived pathogen-associated molecular factors (PAMPs) and effectors, respectively, to activate immune responses. These immune responses include the production of antimicrobial compounds [10] or various pathogenesis-related proteins [11, 12] and the hypersensitive response (HR) [13, 14]. When challenged by HTS, however, plants deploy a distinct subset of sensors [15–17] to perceive the stress and activate thermotolerance responses, which usually include the production of HSPs and antioxidants or reactive oxygen species (ROS) scavengers [18–20].

Despite the distinct natures of plant immunity and thermotolerance, plants employ common signaling components during these processes, such as Ca^2+^, phytohormones including jasmonic acid (JA) and salicylic acid (SA), and various transcription factors (TFs) [21–23]. The existence of these overlapping signaling components hints at the potential for extensive tradeoffs between plant responses to pathogens and to HTS. Indeed, these two processes are thought to be closely related [24]. For example, plant immunity is generally dampened by HTS [25]. Specific defense responses thus require the selective activation of the appropriate shared components. However, how these crucial components regulate specific responses to HTS or pathogen attack remains largely unknown.

The defense responses of plants to either HTS or pathogen attack are largely regulated at the transcriptional level and rely on TFs. Some TFs, such as WRKY25, WRKY33, and CaWRKY40, act as positive regulators of not only plant responses to pathogen attack but also of their responses to HTS [22, 26, 27], indicating that TFs may act as crucial regulators of the tradeoff between plant responses to HTS and pathogen attacks via context-specific targeting. Given that the target genes are widely distributed in the genome and are generally wrapped around histones and compacted into chromatin, their regulation by TFs is likely dependent on their accessibility to the transcriptional machinery through coordinated and ordered local chromatin unwrapping events. Yet how the targeting of these genes by TFs and chromatin unwrapping are coordinately regulated remains to be elucidated.

The chromatin state is believed to be mediated by ATP-dependent chromatin remodeling and histone modifications [28, 29]. ATP-dependent chromatin remodeling is catalyzed by the SWR1 complex (SWR1c) via the replacement of H2A with the histone variant H2A.Z in nucleosomes [30, 31]. The covalent histone modifications methylation and acetylation are regulated by the antagonistic activity of methyltransferases and demethylases [32–34] and of acetyltransferases/deacetylases [35–37], which emerge as complexes such as SET complexes (SET-C) [38] and histone acetyltransferases complexes (HAT-C). NuA3 [39–41] and NuA4 [42] are two classes of HAT-C that function in the acetylation of the histone H3 [43] and of the histones H4, H2A, and H2A.Z, respectively [44, 45]. Each of these complexes comprises multiple members that associate with each other directly or indirectly, and each exhibits different and non-redundant functions [46], indicating that chromatin remodeling and histone modification are complex processes involving the coordinated action of multiple regulatory elements [43]. In addition, these complexes share common subunits. For example, ARP4, SWC4, YAF9, and RUVBL2 have been found in both NuA4-C and SWR1-C [42, 44, 47, 48]. The significance of this sharing of components among different complexes is still not clear.

Perhaps the shared components enable functional cooperation among complexes [42, 49]. For example, SWC4, a homolog of yeast SWR1 complex 4/Esa1-associated factor 2 (Swc4/Eaf2) and human DNA methyltransferase-associated protein 1 (DMAP1) [42, 50], is essential for recruiting SWR1-C to target chromatin regions through the recognition of specific AT-rich DNA elements, thus modulating H2A.Z deposition at key regulatory genes [51]. TAF14b/YAF9A (Transcription initiation factor TFIID subunit 14b/ Protein AF-9 homolog a), which exhibits the highly conserved protein structure YASTS, interacts with acetylated and crotonylated lysine residues in the N-terminal tails of histones. TAF14b-mediated protein-protein interactions are crucial for the formation of the transcription pre-initiation complex [52]. TAF14B physically associates with many critical multi-subunit complexes, including the general transcription factors TFIID and TFIIF; and the chromatin remodeling complexes SWI/SNF, INO80, RSC, and Mediator; and the histone modification enzyme NuA4 and NuA3 [53, 54]. RUVBL1, an evolutionarily highly conserved eukaryotic protein belonging to the AAA(+)-family of ATPases, is shared by INO80, SWR1, and NuA4 [55]. This protein has all the structural characteristics of a molecular motor and even an ATP-driven helicase and has been implicated in chromatin remodeling, DNA repair, and apoptosis [56]. Accumulating evidence that chromatin remodeling via histone modifications such as methylation, acetylation, and ubiquitination, DNA- methylation and histone variant incorporation play important roles in the regulation of pepper immunity and abiotic stress responses [21, 57–61], as well as in the regulation of plant response to heat stress [21, 62–64]. In particular, H2A.Z incorporation and histone modification mediated by SWR1 [46, 65–67] and ISWI [68] have been found to be crucial in regulation of plant immunity, and H2A.Z deposition have been implicated in the regulation of heat stress response [69, 70]. Despite fact that that plants employ a shared set of signaling components in their defense response to attack from pathogens and high temperature stress [21–23], and plant immunity is modulated by high temperature stress, with effector triggered immunity (ETI) being and depressed and PAMP triggered immunity (PTI) being promoted [5, 21, 71], the roles of plant epigenetic regulators such as SWC4, TAF14B and RUVBL1 and how they coordinate in plant immunity and HTS response have not been fully understood, in particular, how the tradeoff between plant immunity and HTS response are regulated at epigenetic level by these regulators remains unclear.

Pepper (*Capsicum annuum*) is one of the most important solanaceous vegetables worldwide. This crop is generally distributed or planted in uplands during the warm season, in subtropical or tropical regions, or in greenhouses. During its growth and development, pepper is frequently exposed to HTS, leading to retarded growth and development. In addition, pepper is often attacked by various soil-borne pathogens such as *R*. *solanacearum*, which has an extremely versatile lifestyle, exclusively invades plants through the roots, and causes bacterial wilt, with heavy losses in productivity [72, 73]. The responses of pepper to attack by *R*. *solanacearum* and HTS appear to be closely linked, since a subset of regulatory proteins, such as CaCDPK15, CaWRKY6, CaWRKY27b, CaWRKY40, CabZIP63, CaNAC2c and CaHsfB2a are involved in responses to both stresses[27, 74–79], pointing to a tradeoff between these two responses. In the present study, we provide evidence CaSWC4 acts as a positive regulator in pepper response to RSI and to HTS by recruiting CaWRKY40 and CabZIP63 via physical interaction and brings them to their targets (immunity- or thermotolerance-related genes) by binding to AT-rich DNA elements in their promoters and promoting deposition of the active chromatin marks H2A.Z, H3K9ac, and H4K5ac on the target chromatin by association with CaTAF14 and CaRuvBL2 in context dependent manner, in this way, immunity and thermotolerance are rapidly and accurately activated by CaWRKY40 and CabZIP63 upon RSI and HTS, respectively.

## Results

### *CaSWC4* is targeted by CaWRKY40 and upregulated by RSI and HTS

We previously demonstrated that both CabZIP63 and CaWRKY40 positively regulate RSI resistance and thermotolerance in pepper, with *CaWRKY40* being directly targeted and regulated by CabZIP63. To clarify how CabZIP63 and CaWRKY40 balance plant immunity and thermotolerance, we comprehensively analyzed RNA-seq data (data have been deposited into CNGB: https://db.cngb.org/search/?q=CNP0001104) and performed chromatin immunoprecipitation sequencing (ChIP-seq) using *CaWRKY40-GFP*-overexpressing pepper plants challenged with RSI (S1A and S1B Fig and S3 Table). Among a total of 1788 potential targets of CaWRKY40 (with threshold q-value = 0.05), a candidate gene encoding SWC4 (named CaSWC4) aroused our interest, since it is homologous to yeast Swc4/Eaf2 and human DMAP1 proteins [80, 81] and might be shared by two chromatin-modifying complexes: the SWR1 complex (SWR1-C) and the NuA4 acetyltransferase complex [82], and SWR1-C is crucial for the regulation of chromatin remodeling during plant immunity against pathogens [46, 65], but its direct association with transcription factors in the coordination of plant immunity and thermotolerance remains under-investigated. To test this possibility, we examined the promoter sequence of *CaSWC4* based on sequencing data. We identified a typical W-box in this promoter, and found that CaWRKY40 was enriched in the *CaSWC4* promoter upon challenge by RSI or HTS, as revealed by ChIP-qPCR (S1C Fig). *CaSWC4* was upregulated by transient overexpression of *CaWRKY40* in the leaves of pepper plants challenged with RSI or HTS (S1D Fig). Similar to *CaWRKY40*, *CaSWC4* was upregulated by RSI from 3 to 12 hpi and by HTS challenge from 3 to 48 hpt in pepper plants, as revealed by reverse transcription quantitative polymerase chain reaction (RT-qPCR) (S1E Fig). These results suggest that CaSWC4 might be involved in plant immunity against RSI and in thermotolerance.

### CaSWC4 is nuclear protein that positively regulates plant responses to RSI and HTS

To determine the subcellular localization of CaSWC4, we transiently expressed this protein fused to yellow fluorescent protein (YFP) in *Nicotiana benthamiana* leaves via agroinfiltration. The CaSWC4-YFP fusion protein (driven by the 35S promoter) targeted the nucleus, whereas YFP alone was detected throughout the cell, including the nucleus, cytoplasm, and plasma membrane (S2 Fig). To explore the role of CaSWC4 in the responses to RSI and HTS, we transiently overexpressed this protein (CaSWC4-TO) in pepper leaves (S3A and S3B Fig). Transient overexpression of CaSWC4-YFP induced clear HR mimic cell death, including darker trypan blue staining, greater ion leakage (revealed by a higher levels of leaf relative conductivity), and higher H_2_O_2_ accumulation [revealed by darker diaminobenzidine (DAB) staining] than in the control (S3C and S3F Fig). CaSWC4 overexpressing pepper leaves were less affected by HTHH than control plants, as seen by lower Fv/Fm and photosystem II (PSII) photochemical efficiency in the light (фPSII) (S3D and S3E Fig). In addition, the immunity-related marker genes *CaNPR1* and *CaDEF1* and the thermotolerance marker gene *CaHSP24* were upregulated in leaves transiently overexpressing CaSWC4-YFP (S3G Fig). These results indicate that CaSWC4 positively regulates plant immunity and thermotolerance.

**Fig 1:**
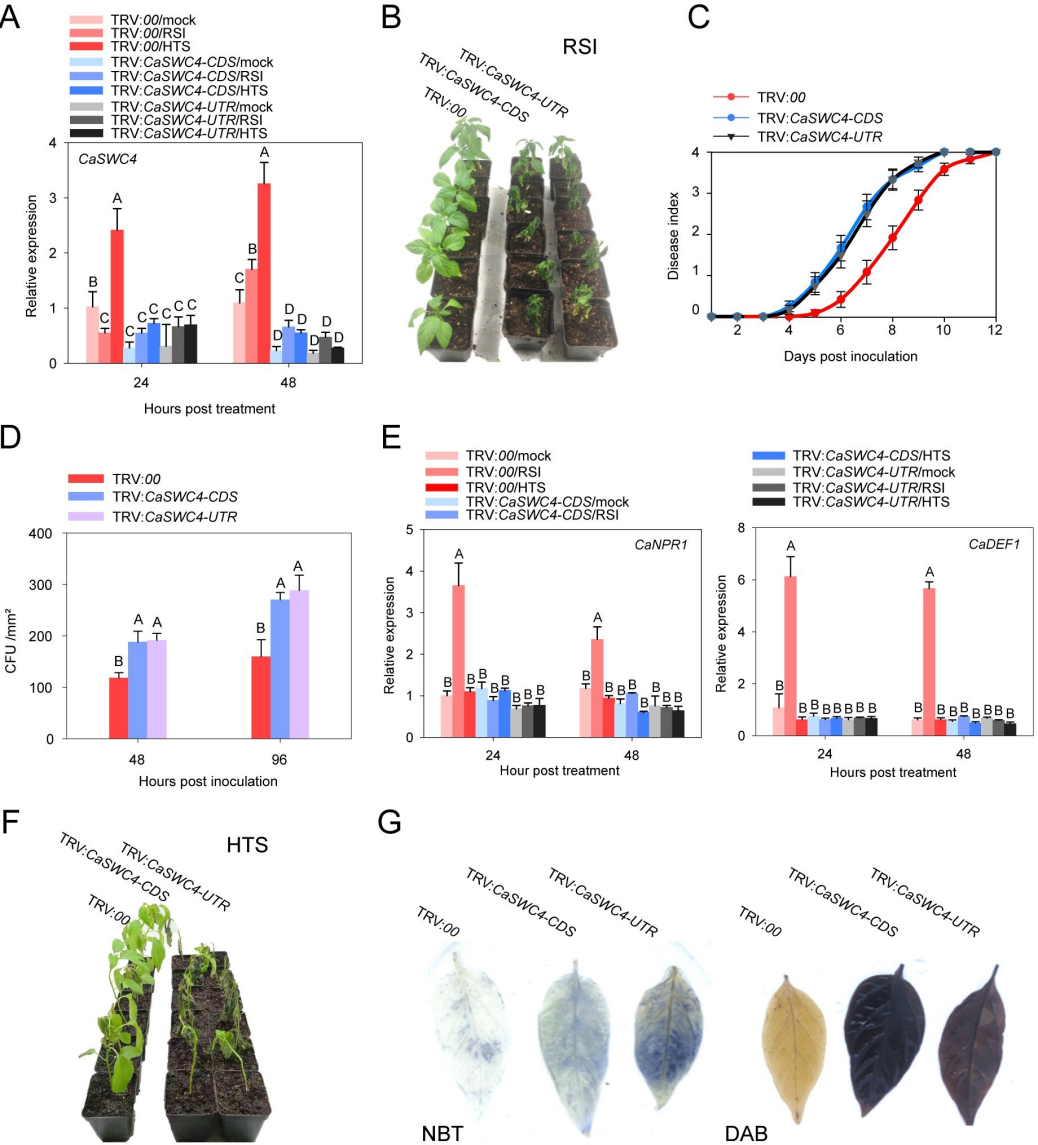
Phenotypes of *CaSWC4*-silenced pepper plants include thermotolerance and resistance to RSI. **A,** Relative transcript levels of *CaSWC4* in *CaSWC4*-silenced pepper challenged with RSI at room temperature (28°C, 90% relative humidity), with HTS (42°C, 90% relative humidity), or mock treated (28°C, no RSI). **B**, Phenotypic effect of *CaSWC4* silencing on plant resistance to RSI. **C-D,** *R*. *solanacearum*-inoculated, *CaSWC4*-silenced plants showed higher disease indices (S2 Table) and pathogen growth than the wild type (shown as colony-forming units [cfu]). **E,** Relative transcript levels of the immunity-associated genes *CaNPR1* and *CaDEF1* in pepper plants (*CaSWC4*-silenced and control) challenged by *R*. *solanacearum* infection and HTS. **F,** *CaSWC4*-silenced pepper plants displayed reduced thermotolerance. **G,** H_2_O_2_ and ROS accumulation, as observed by DAB and NBT staining, in leaves of *CaSWC4*-silenced pepper plants challenged with HTS. Data presented are means ± standard error (SE) of four replicates. In A, D, and E, different letters indicate significant differences among means (*P* < 0.01), as determined by Fisher’s protected LSD test.

To confirm these findings, we silenced *CaSWC4* in pepper plants by virus-induced gene silencing (VIGS). *CaSWC4* transcript levels were greatly reduced in plants expressing two distinct vectors targeting specific fragments in the coding sequence and 3’ UTR of *CaSWC4* mRNA (Fig 1A). To investigate the role of *CaSWC4* in the response to RSI, we inoculated TRV:00 control plants and *CaSWC4*-silenced pepper plants with *R*. *solanacearum*. The silencing of *CaSWC4* significantly reduced plant resistance to RSI, as the *CaSWC4*-silenced plants had higher disease indices from 4 to 12 dpi and higher levels of *R*. *solanacearum* growth than the wild type (Fig 1B–1D). Consistent with this, silencing *CaSWC4* significantly reduced the transcript levels of immunity-related marker genes, including *CaNPR1* and *CaDEF1*, during the plant response to RSI (Fig 1E). The silencing of *CaSWC4* also significantly weakened plant thermotolerance and increased ROS accumulation [darker DAB and nitro blue tetrazolium (NBT) staining] in response to HTS compared to those in control plants (Fig 1F–1G). In a complementary approach, we generated *N*. *benthamiana* lines overexpressing *CaSWC4* (*CaSWC4*-OE). In contrast to pepper plants silenced for *CaSWC4*, these *N*. *benthamiana CaSWC4*-OE plants were more resistant to RSI and more tolerant of HTS than the controls (S4 Fig). These results indicate that CaSWC4 positively regulate the responses of pepper to RSI and HTS by interacting with CabZIP63 and CaWRKY40, thereby recruiting probably these transcription factors to their target genes.

### CabZIP63 and CaWRKY40 are functionally dependent on CaSWC4 during plant responses to RSI and HTS

**Fig 2:**
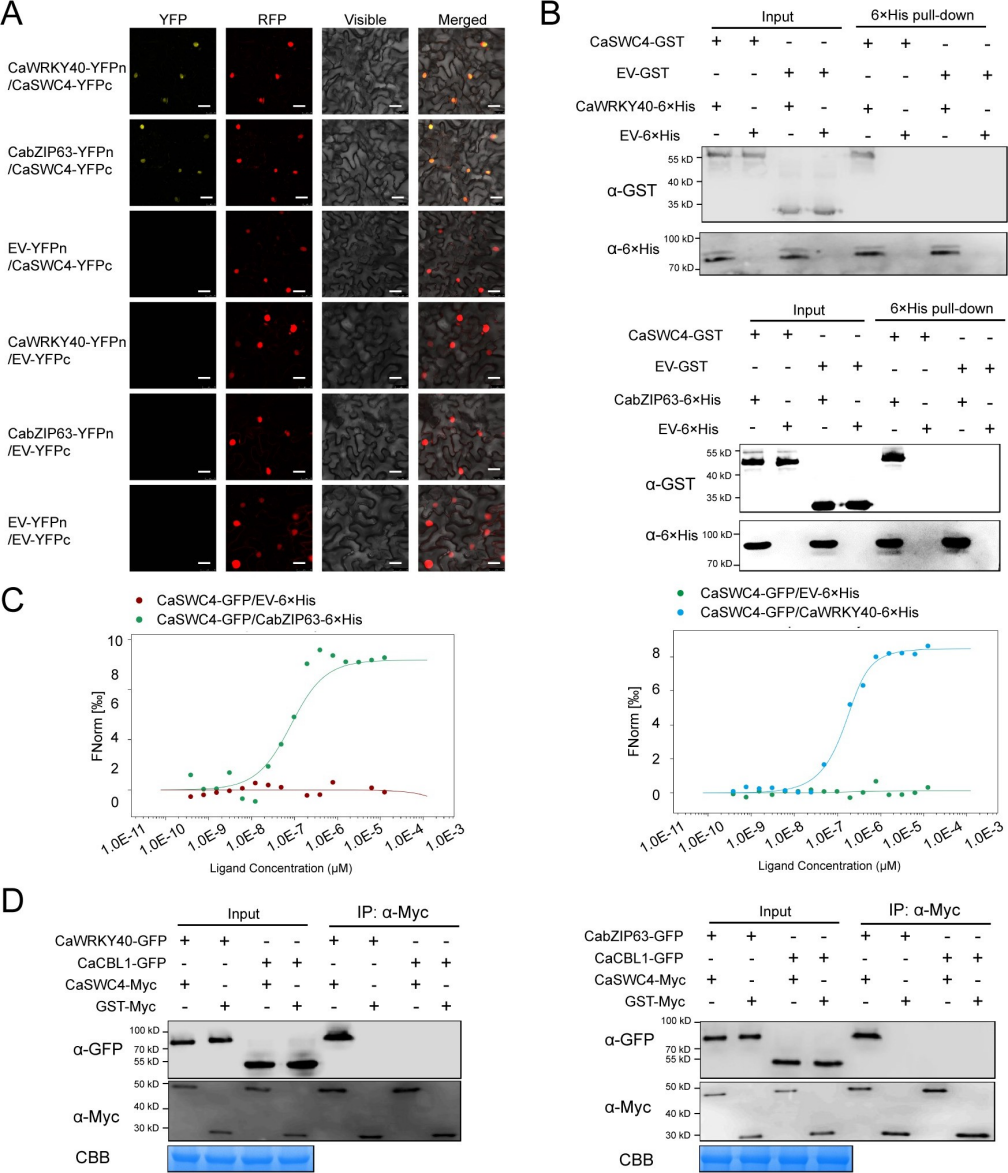
CaSWC4 interacts with CaWRKY40/CabZIP63 *in vivo* and *in vitro*. **A,** BiFC confirmation of the interaction between CaWRKY40/CabZIP63 and CaSWC4 in *N*. *benthamiana* leaves infiltrated with *Agrobacterium* GV3101 cells bearing *CaSWC4-YFP*^*C*^+ *CaWRKY40/CabZIP63-YFP*^*N*^. NbH2B (histone H2B)-RFP was used to indicate the nucleus. Red fluorescence and yellow fluorescence, visible light and merged images were taken under confocal microscope at 48 hpi. Bars = 25 μm. **B,** Pull-down assay revealing the *in vitro* interaction between CaSWC4 and CaWRKY40/CabZIP63. CaSWC4-GST was incubated with CaWRKY40/CabZIP63-6×His and Ni Smart beads for 1 h at 4°C with slow rotation. The bound proteins were eluted from the beads and detected using anti-His or anti-GST antibody. **C,** Analysis of the *in vitro* interaction between CaSWC4 and CaWRKY40/CabZIP63 by MST. CaSWC4-GFP: target; CaWRKY40/CabZIP63/EV-6×His: ligand. Mixtures of GFP and CaWRKY40/CabZIP63-6×His or CaSWC4-GFP and CaWRKY40/CabZIP63-6×His were loaded onto Monolith NT.115 capillaries and measured using 50% IR laser power and an LED excitation source with λ = 470 nm at ambient temperature. **D,** Interaction between CaSWC4 and CaWRKY40/CabZIP63 *in vivo*, as determined by Co-IP assay. Proteins were isolated from pepper leaves transiently overexpressing *CaSWC4-Myc* and *CaWRKY40/CabZIP63-GFP*, and CaSWC4-Myc was immunoprecipitated with anti-Myc antibody (CaCBL1-GFP or GST-Myc was used as a negative control).

As CaSWC4, CaWRKY40, and CabZIP63 show similar transcriptional expression patterns and functions during plant responses to RSI and HTS, we reasoned that these three proteins might be physically related. To test this hypothesis, we assayed their possible interactions by bimolecular fluorescence complementation (BiFC) in *N*. *benthamiana* leaves. Both CabZIP63 and CaWRKY40 interacted with CaSWC4 in the nucleus. We further confirmed these interactions by microscale thermophoresis (MST), pull-down, and co-immunoprecipitation (Co-IP) assays. By contrast, CaCBL1, which was used as a negative control, did not interact with CaSWC4. These results indicate that CaWRKY40 and CabZIP63 bind to CaSWC4 (Fig 2).

To better understand the functional relationships of these proteins during plant responses to RSI and HTS, we transiently overexpressed *CaWRKY40* or *CabZIP63* (CaWKRY40-TO or CabZIP63-TO) in wild type (control) and *CaSWC4*-silenced pepper plants to assay the effect of *CaSWC4* silencing on the induction of immunity- or thermotolerance-related genes by CaWKRY40 or CabZIP63. In wild type plants, the immunity- or thermotolerance-related genes *CaDEF1*, *CaNPR1*, and *CaHSP24* were significantly upregulated by *CaWRKY40* overexpression, and *CaWRKY40* was upregulated by *CabZIP63* overexpression, as observed in our previous studies (Dang, 2013; Shen, 2016). However, the upregulation of these genes was significantly blocked by *CaSWC4* silencing (S5 Fig). Consistent with this, the transcript levels of *CabZIP63*, which forms a positive feedback loop with *CaWRKY40*, were also reduced by *CaSWC4* silencing (S5A Fig). HR-like cell death induced by transient overexpression of *CaWRKY40* or *CabZIP63* was significantly blocked by the silencing of *CaSWC4* (S5C Fig).

**Fig 3:**
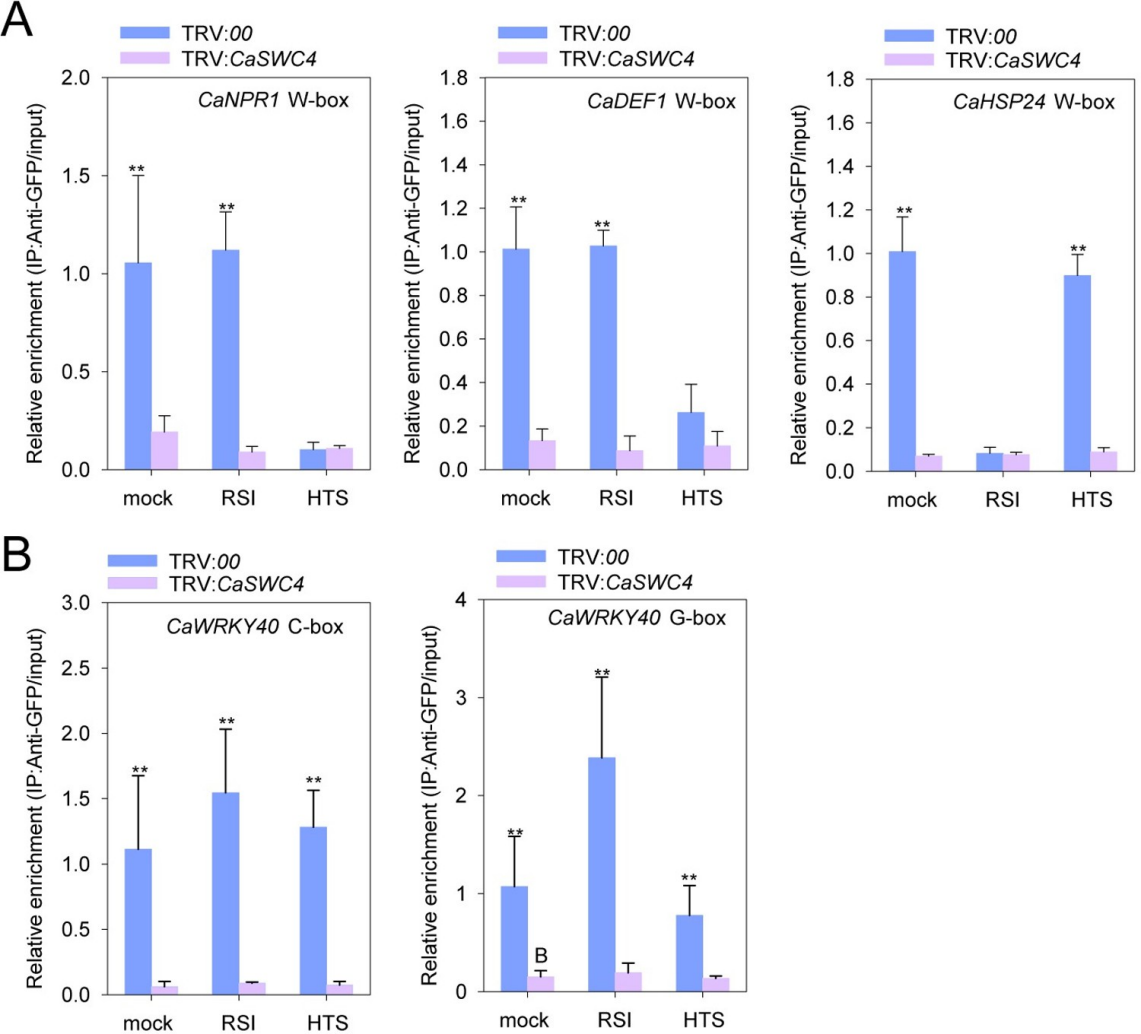
*CaSWC4* silencing reduces the binding of CaWRKY40 and CabZIP63 to the W-box and G/C-boxes in the promoters of their target genes, respectively. **A.** Relative enrichment of CaWRKY40-GFP in the W-box-containing promoters of *CaNPR1*, *CaDEF1*, and *CaHSP24* in the leaves of *CaSWC4*-silenced pepper plants transiently overexpressing *CaWRKY40-GFP*, as revealed by ChIP-qPCR. **B.** Relative enrichment of CabZIP63-GFP at the C- or G-box containing *CaWRKY40* promoter in the leaves of *CaSWC4*-silenced pepper plants transiently overexpressing *CabZIP63-GFP*, as revealed by ChIP-qPCR. Data are shown as means ± standard error of three replicates, asterisks above the bars indicated significant differences among means (P < 0.01), as calculated with t-test.

In parallel, we assayed the effect of *CaSWC4* silencing on the enrichment of CaWRKY40 at the *CaNPR1*, *CaDEF1*, and *CaHSP24* loci by ChIP-qPCR. CaWRKY40 exhibited a higher level of enrichment around the W-boxes in the *CaNPR1* and *CaDEF1* promoters when control plants were challenged with RSI. CaWRKY40 also showed greater enrichment around the W-box in the *CaHSP24* promoter in control plants challenged by HTS. In both cases, however, this enrichment was significantly reduced by silencing of *CaSWC4* (Fig 3A). Likewise, the enrichment of CabZIP63 around the C- or G-box in the *CaWRKY40* promoter was also reduced by *CaSWC4* silencing (Fig 3B).

**Fig 4:**
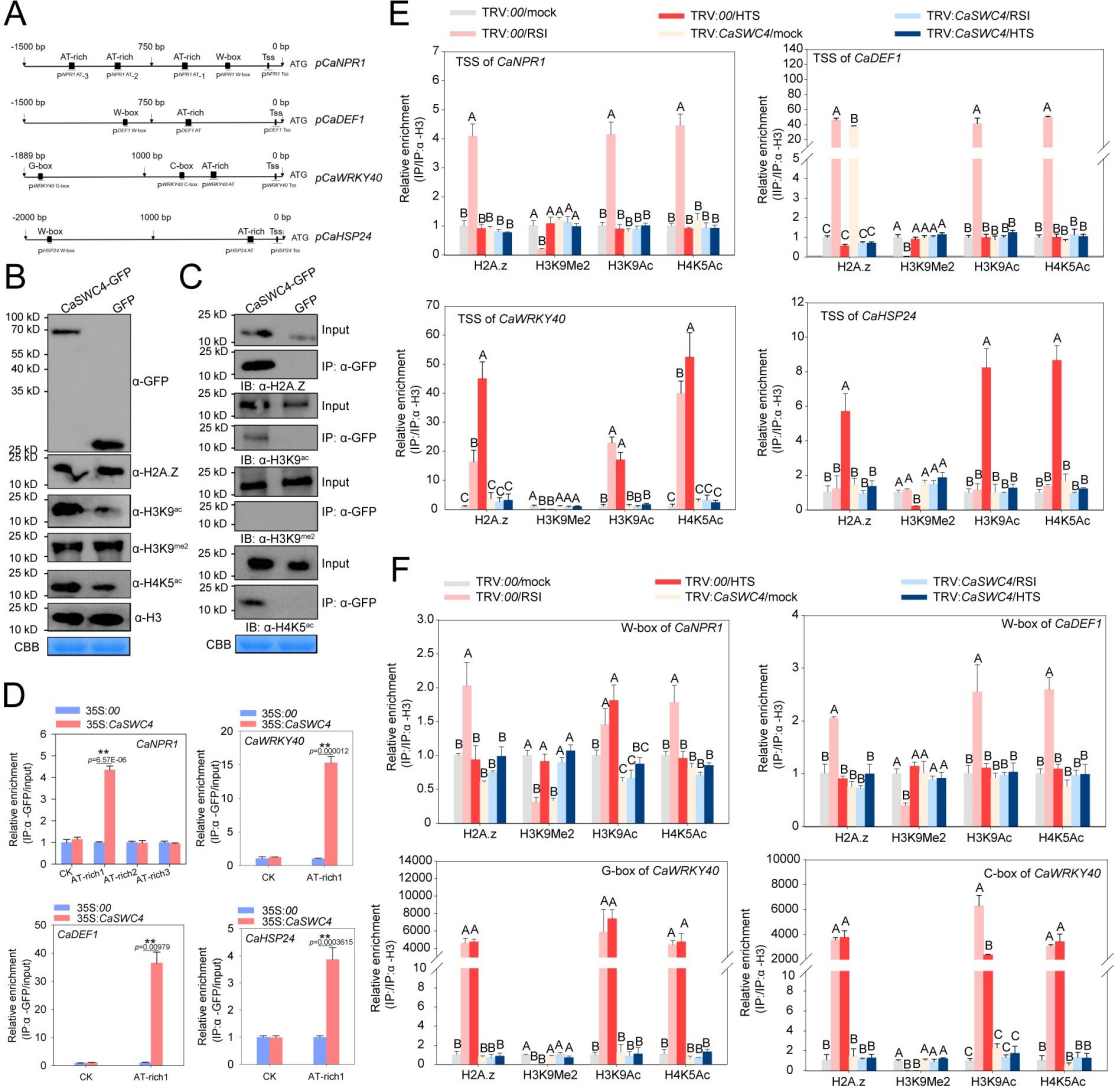
CaSWC4 binds to and regulates the accumulation of H3K9Me2, H2A.Z, H3K9ac, and H4K5ac around the promoters of *CaNPR1*, *CaDEF1*, *CaWRKY40*, and *CaHSP24*. **A,** Schematic diagram of the G-box, C-box, W-box, AT-rich, and TSS cis-elements in the promoters of *CaWRKY40*, *CaNPR1*, *CaDEF1*, and *CaHSP24*. Black blocks represented the G-box, C-box, W-box, AT-rich element, and TSS. The names of primer pairs used to amplify the target DNA fragments in the promoters are underlined. **B,** Immunoblot analysis and quantification of H3K9Me2, H2A.Z, H3K9ac, and H4K5ac levels in total extracts from leaves transiently overexpressing *CaSWC4-YFP* and *YFP*. **C,** Interaction between CaSWC4 and H3K9me2, H2A.Z, H3K9ac, and H4K5ac *in vivo*, as determined by Co-IP assay. Proteins were isolated from pepper leaves transiently overexpressing *CaSWC4-YFP*, and CaSWC4-YFP was immunoprecipitated with anti-YFP antibody. The bound proteins were eluted from the beads and detected using an anti-H3K9Me2, anti-H2A.Z, anti-H3K9ac, and anti-H4K5ac antibodies. **D,** CaSWC4 directly targets the *CaWRKY40*, *CaNPR1*, *CaDEF1*, and *CaHSP24* promoters, as shown by ChIP-qPCR. Chromatin was isolated from pepper leaves transiently overexpressing *CaSWC4-YFP* sheared into 300–500 bp fragments. The DNA was immunoprecipitated with antibodies against GFP, and purified DNA was used as template with a specific primer pair targeting the AT-rich promoter region. Data are shown as means ± standard error of three replicates, asterisks above the bars indicated significant differences among means (P < 0.01), as calculated with t-test. **E-F,** Relative enrichment of H3K9Me2, H2A.Z, H3K9ac, and H4K5ac within the TSSs (enrichment of histone 3 as internal), W-box, or G/C-box in the promoters of *CaWRKY40*, *CaNPR1*, and *CaDEF1* upon RSI or HTS treatment in control or *CaSWC4*-silenced plants, as determined by ChIP-qPCR. Data presented are means ± standard error (SE) of four replicates. In E and F, different letters indicate significant differences among means (*P* < 0.01), as determined by Fisher’s protected LSD test. RSI: *Ralstonia solanacearum* infection, HTS: high temperature stress.

SWC4 recognizes and binds to specific AT-rich DNA elements in the chromatin regions of its target genes [51]. The binding of CaSWC4 to AT-rich DNA elements was corroborated by electrophoretic mobility-shift assays (EMSAs) (S6 Fig). To investigate whether CaSWC4 binds to DNA around AT-rich DNA elements in the promoters of *CaNPR1*, *CaDEF1*, *CaWRKY40*, and *CaHSP24* in vivo, we conducted ChIP-qPCR analysis of CaSWC4-TO pepper plants, which showed that CaSWC4 was significantly enriched around AT-rich DNA elements containing the promoter regions of *CaNPR1*, *CaDEF1*, *CaWRKY40*, and *CaHSP24* (Fig 4A and 4D). The binding of CaSWC4 to AT-rich DNA elements may contribute to the recruitment of transcription factor such as CaWRKY40 and CabZIP63 to certain target genes by recognizing specific AT-rich sequences. These data indicate that the targeting and transcriptional regulation of immunity-related genes and thermotolerance-related genes by CaWRKY40 and CabZIP63 are dependent on CaSWC4.

### CaSWC4 modulates the targeting of CabZIP63 and CaWRKY40 during plant responses to RSI and HTS by orchestrating chromatin remodeling

SWC4 is shared by the SWR1 complex (SWR1-C) and the NuA4 acetyltransferase complex, which are crucial regulators of H2A.Z exchange and of histone acetylation during chromatin remodeling [83, 84]. Therefore, to determine whether CaSWC4 promotes chromatin remodeling of the target genes of CaWRKY40 or CabZIP63, we investigated the effects of CaSWC4-TO on the accumulation of chromatin-activation-related H2A.Z, H3K9ac, and H4K5ac as well as chromatin-inactivation-related H3K9me2 marks [53]. CaSWC4-TO enhanced the accumulation of H2A.Z, H3K9ac, and H4K5ac in pepper leaves but did not affect H3K9me2 accumulation (Fig 4B). In addition, we assayed the interaction of CaSWC4 with H2A.Z, H3K9ac, H4K5ac, and H3K9me2 by immunoblotting and found that CaSWC4 interacted with antibodies against all of these proteins except H3K9me2 (Fig 4C).

We then assayed the effect of CaSWC4-TO on chromatin remodeling in loci harboring immunity- or thermotolerance-related genes by detecting the enrichment of chromatin-activation-related H2A.Z, H3K9ac, and H4K5ac and chromatin-inactivation-related H3K9me2 marks. RSI treatment led to significantly enhanced deposition of H2A.Z, H3K9ac, and H4K5ac around the TSS or W-boxes in the *CaNPR1* and *CaDEF1*, but significantly reduced deposition of H3K9me2 (Fig 4E). Similarly, HTS treatment resulted in significantly enhanced deposition of H2A.Z, H3K9ac, and H4K5ac around the TSS and W-box in the promoter of the thermotolerance-related gene *CaHSP24* and significantly reduced deposition of H3K9me2. Moreover, the deposition of H2A.Z, H3K9ac, and H4K5ac around the TSS, G-box, or C-box in the *CaWRKY40* promoter was significantly enhanced, and that of H3K9me2 was significantly reduced, by either RSI or HTS (Fig 4F). However, these processes were significantly blocked by *CaSWC4* silencing during plant responses to RSI or HTS (Fig 4E and 4F). These data are closely related to the finding that neither immunity- nor thermotolerance-related genes were upregulated by RSI or HTS in *CaSWC4-*silenced pepper plants. These results indicate that CaSWC4 contributes to the regulation of CaWRKY40- and CabZIP63-targeted immunity- and thermotolerance-related genes via chromatin remodeling during plant responses to RSI and HTS.

### CaTAF14b and CaRuvBL2 interact with CaSWC4 to positively regulate plant responses to RSI or HTS

Our data indicate that the immunity-related genes *CaNPR1* and *CaDEF1* are specifically activated in pepper upon RSI, the thermotolerance-related gene *CaHSP24* is specifically activated in plants challenged with HTS, and *CaWRKY40* is activated under both conditions. Consistent with this, the CaSWC4-mediated deposition of H2A.Z, H3K9ac, H4K5ac, and H3K9me2 around the promoters of thermotolerance-related genes was inhibited during plant responses to RSI. In addition, CaSWC4-mediated deposition of chromatin marks around the promoters of immunity-related genes was inhibited during plant responses to HTS (Fig 4E and 4F). However, the intensity of the binding of SWC4 to AT-rich DNA elements in the promoters of immunity- or thermotolerance- related genes was not affected during plant responses to either stress (S7 Fig). These findings imply that CaSWC4 might rapidly and accurately modulate the chromatin remodeling of immunity- or thermotolerance-related genes by recruiting SWR1 or other members of the NuA4 complex to help maintain the balance between immunity and thermotolerance.

**Fig 5:**
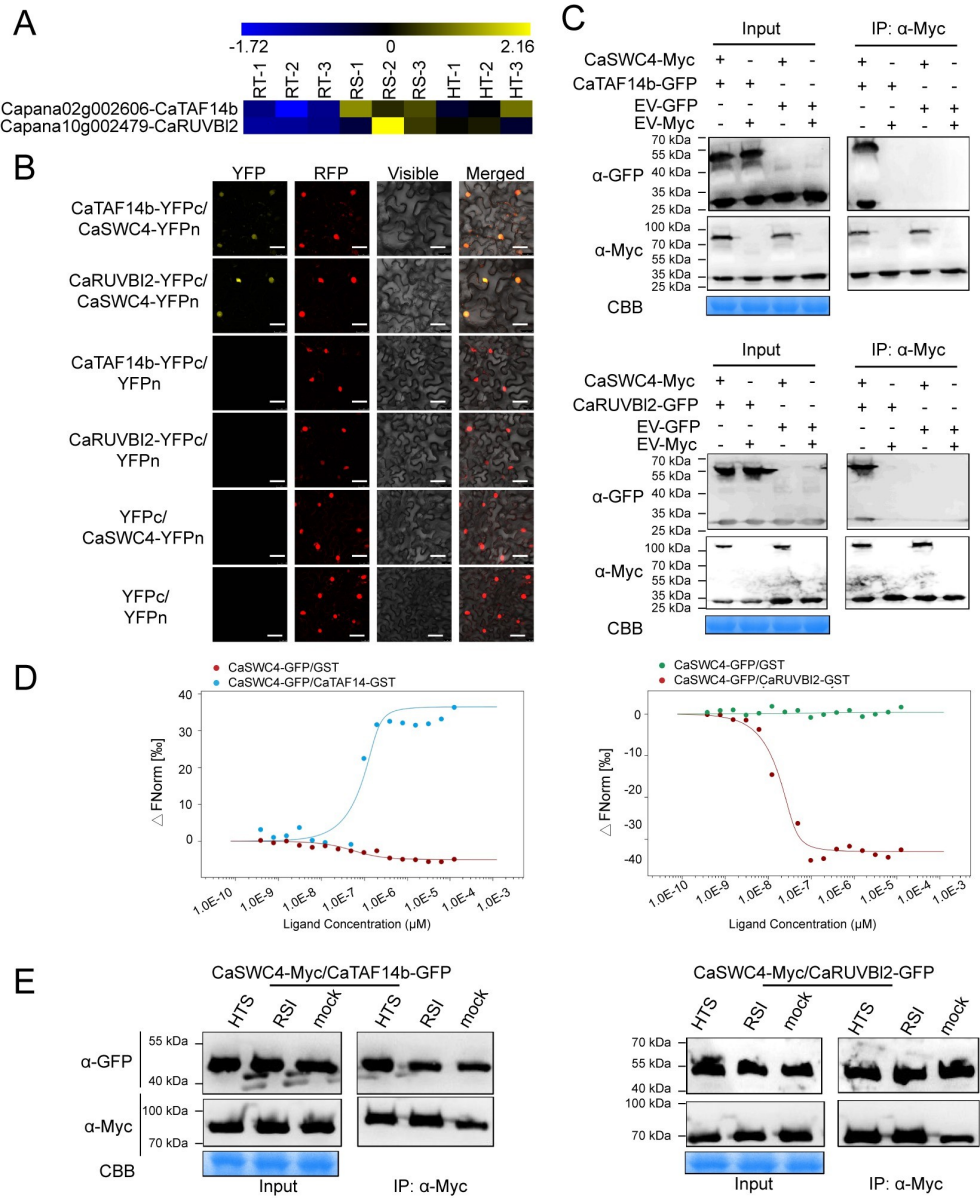
CaSWC4 interacts with CaTAF14b and CaRUVBL2 *in vivo* and *in vitro*. **A,** FPKM (Fragments per Kilobase Million) of *CaTAF14b* and *CaRUVBL2* in pepper plant challenged with HTS or RSI from the RNA-seq data. **B,** BiFC confirmation of the interaction between CaTAF14/CaRUVBL2 and CaSWC4 in *N*. *benthamiana* leaves infiltrated with *Agrobacterium* GV3101 cells bearing CaTAF14b/CaRUVBL2*-YFP*^*C*^ + CaSWC4*-YFP*^*N*^. NbH2B (histone H2B)-RFP was used to indicate the nucleus. Red fluorescence and yellow fluorescence, visible light and merged images were taken under a confocal microscope at 48 hpi. Bars = 25 μm. **C,** Interaction between CaSWC4 and CaTAF14b/CaRUVBL2 *in vivo*, as determined by Co-IP assay. Proteins were isolated from pepper leaves transiently overexpressing *CaSWC4-Myc* and CaTAF14b/CaRUVBL2*-GFP*, and CaSWC4-Myc was immunoprecipitated with anti-Myc antibody. **D,** Analysis of the interaction *in vitro* between CaSWC4 and CaTAF14/CaRUVBL2 by MST. GFP or CaTAF14b/CaRUVBL2-GFP: target; CaSWC4-6×His: ligand. Mixtures of GFP/CaSWC4-6His or CaTAF14b/CaRUVBL2-GFP/CaSWC4-6×His were loaded onto Monolith NT.115 capillaries and measured using 50% IR laser power and an LED excitation source with λ = 470 nm at ambient temperature. **E,** Analysis of the interaction strength between CaTAF14b/CaRUVBL2 and CaSWC4, as determined by quantitative Co-IP assay. Proteins (500 ng loaded) were isolated from pepper leaves transiently overexpressing *CaSWC4-Myc* and CaTAF14b/CaRUVBL2*-GFP* challenged with RSI and HTS, and CaSWC4-Myc was immunoprecipitated with anti-Myc antibody.

**Fig 6:**
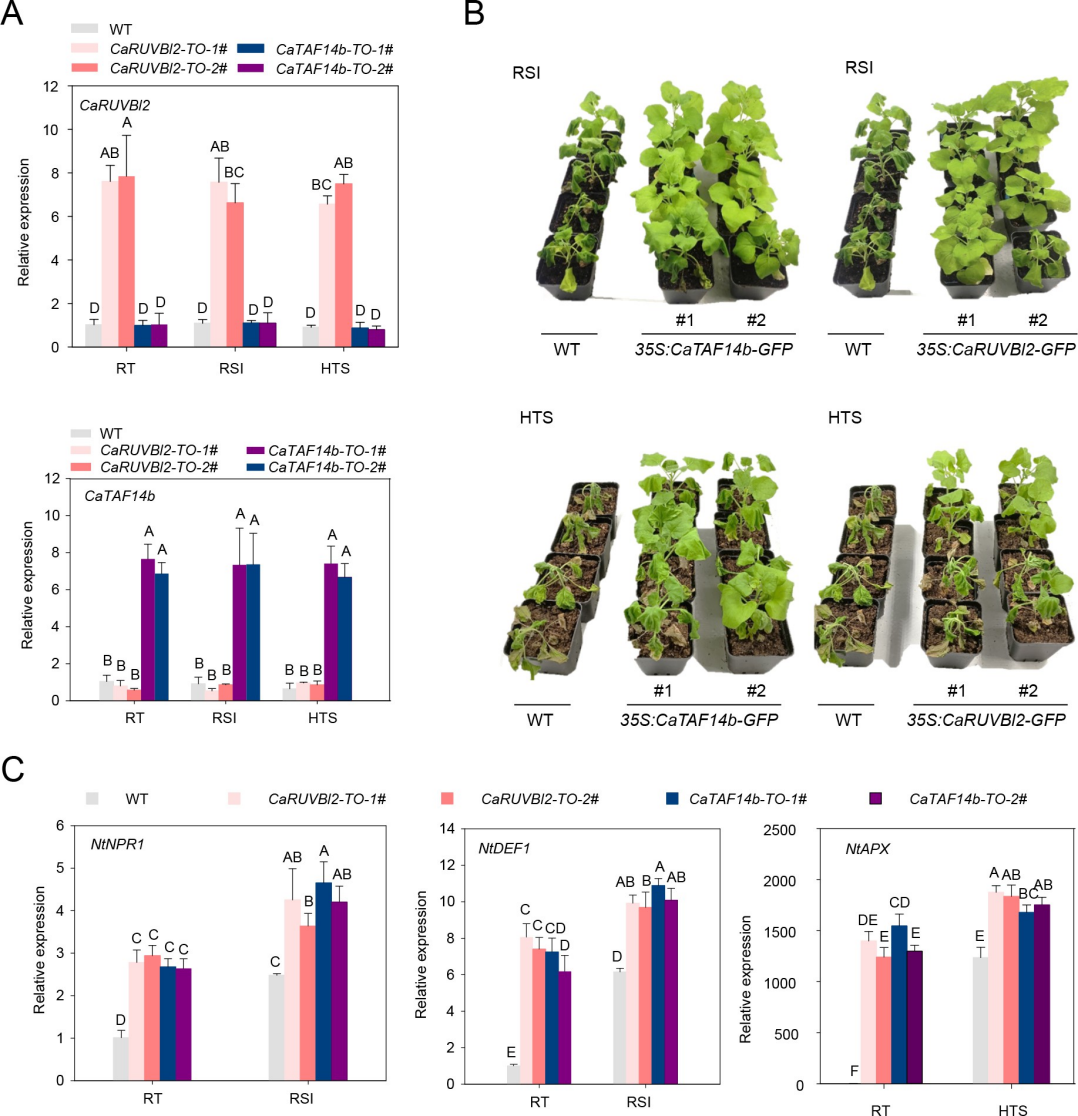
Heterologous overexpression of *CaTAF14b* or *CaRUVBL2* in *N*. *benthamiana* plants results in increased tolerance of RSI and HTS. **A,** *CaTAF14b/CaRUVBL2* transcript levels in two T_2_ transgenic *N*. *benthamiana* lines challenged with RSI and HTS. **B,** *N*. *benthamiana* plants overexpressing *CaTAF14b/CaRUVBL2* displayed increased thermotolerance and resistance to *R*. *solanacearum* inoculation. **C,** The immunity-associated genes *NtNPR1* and *NtDEF1* and the thermotolerance-related gene *NtAPX* were transcriptionally upregulated in *N*. *benthamiana* plant overexpressing *CaTAF14b/CaRUVBL2* and challenged by RSI and HTS. Data presented are means ± standard error (SE) of four replicates. In A and C, different letters indicate significant differences among means (*P* < 0.01), as determined by Fisher’s protected LSD test.

To test this hypothesis, we conducted deep transcriptome (RNA-seq) analysis of pepper plants under RSI or HTS treatment and determined that SWR1-C members *CaTAF14b* and *CaRUVBL2* were significantly induced by RSI or HTS (data have been deposited into CNGB: https://db.cngb.org/search/?q=CNP0001104) (Fig 5A). We tested the interaction between CaSWC4 with CaTAF14b or CaRUVBL2 and found that CaSWC4 interacted with both proteins in the nucleus (Fig 5B–5E). In addition, both *CaTAF14b* and *CaRUVBL2* were upregulated by HTS or RSI treatment; however, silencing of *CaTAF14b* or *CaRUVBL2* prevented this transcriptional induction. These results indicate that the silencing of *CaTAF14b* and *CaRUVBL2* in pepper plants was successful and that the transcriptional induction of *CaTAF14b* and *CaRUVBL2* by RSI and by HTS is interdependent (S8A Fig). In addition, silencing of either *CaTAF14b* or *CaRUVBL2* significantly reduced pepper resistance to RSI and thermotolerance coupled with the downregulation of *CaWRKY40* (S8B Fig). By contrast, when we generated two T_2_ lines of *N*. *benthamiana* plants with high *CaTAF14b* or *CaRUVBL2* expression levels (Fig 6A), we found that the overexpression of either gene significantly enhanced the transcript levels of both immunity- and thermotolerance-related genes, including *NtNPR1*, *NtDEF1* and *NtAPX* (Fig 6C). Consistent with this, transgenic plants harboring *CaTAF14b* or *CaRUVBL2* showed significantly enhanced resistance to RSI and HTS compared to wild-type plants (Fig 6B).

### CaSWC4-mediated chromatin remodeling is dependent on CaTAF14b or CaRUVBL2

As CaSCW4 physically interacts with CaTAF14b or CaRUVBL2, to assay the functional relationship between *CaSWC4* and *CaTAF14b* or *CaRUVBL2*, we transiently overexpressed *CaSWC4* in *CaTAF14b-* or *CaRUVBL2-*silenced and control pepper leaves and assayed HR-like cell death. *CaSWC4* was upregulated by RSI and by HTS, but this upregulation was reduced by silencing either *CaTAF14b* or *CaRUVBL2* (S10A Fig). In addition, the CaSWC4-induced upregulation of *CaNPR1*, *CaDEF1*, and *CaHSP24* was blocked by silencing of either *CaTAF14b* or *CaRUVBL2* (S10C Fig). Likewise, transiently overexpression of *CaSWC4* triggered a significant level of HR-like cell death, as manifested by enhanced ion leakage (higher levels of leaf conductivity), but this response was significantly inhibited by the silencing of *CaTAF14b* or *CaRUVBL2* (S9 Fig). These data imply that CaSWC4 is functionally dependent on CaTAF14b and CaRUVBL2 during plant responses to RSI and HTS.

**Fig 7:**
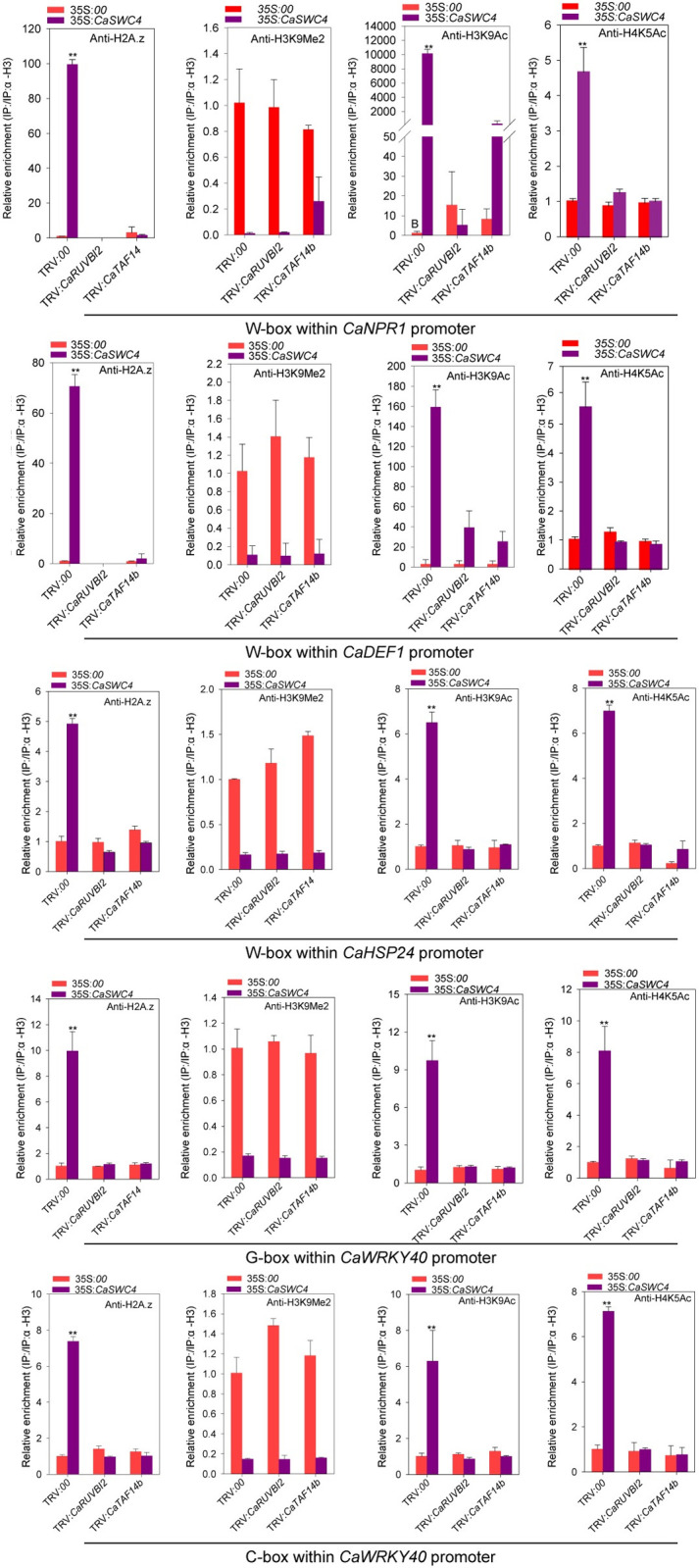
CaSWC4-mediated accumulation of H3K9Me2, H2A.Z, H3K9ac, and H4K5ac around the promoters of target genes requires CaTAF14b/CaRUVBL2. Relative enrichment of H3K9Me2, H2A.Z, H3K9ac, and H4K5ac (enrichment of histone 3 as internal) within the W-box or G/C-box in the *CaWRKY40*, *CaNPR1*, *CaDEF1*, and *CaHSP24* promoters in the leaves of *CaTAF14b/CaRUVBl2*-silenced plants, control plants, or plants transiently overexpressing *CaSWC4-YFP*, as determined by ChIP-qPCR. Data are shown as means ± standard error of three replicates, asterisks above the bars indicated significant differences among means (P < 0.01), as calculated with t-test.

In addition, to determine whether the CaSWC4-modulated chromatin remodeling of the targets of CabZIP63 and CaWRKY40 might be related to CaTAF14b or CaRUVBL2, we assayed the effect of *CaTAF14b* or *CaRUVBL2* silencing on chromatin remodeling activated by CaSWC4-TO. CaSWC4-enhanced H2A.Z, H3K9ac, and H4K5ac deposition around the TSSs or W-boxes in the *CaNPR1*, *CaDEF1*, and *CaHSP24* promoters was blocked by silencing either *CaTAF14b* or *CaRUVBL2*. By contrast, the effect of CaSWC4 in reducing H3K9me2 deposition around TSSs or W-boxes in the *CaNPR1*, *CaDEF1*, and *CaHSP24* promoters was not affected by the silencing of either *CaTAF14b* or *CaRUVBL2*. In addition CaSWC4-enhanced H2A.Z, H3K9ac, and H4K5ac deposition around the TSSs, G-box, or C-box in the *CaWRKY40* promoter was blocked by silencing of either *CaTAF14b* or *CaRUVBL2*, whereas the CaSWC4-induced reduction of H3K9me2 deposition around the G-box or C-box in the *CaWRKY40* promoter was not affected by the silencing of either *CaTAF14b* or *CaRUVBL2* (Figs 7 and S10B). These results indicate that CaSWC4-enhanced chromatin activation via H2A.Z, H3K9ac, and H4K5ac deposition is dependent on CaTAF14b and CaRUVBL2, whereas the CaSWC4-induced reduction in chromatin inactivation-related H3K9me2 deposition is not dependent on these proteins. Consequently, the CaSWC4-induced upregulation of *CaNPR1*, *CaDEF1*, *CaHSP24*, and *CaWRKY40* was blocked by silencing of either *CaTAF14b* or *CaRUVBL2* (S10C Fig).

### CaSWC4 recruits CaTAF14b and CaRUVBL2 to different targets during plant responses to RSI or HTS

**Fig 8:**
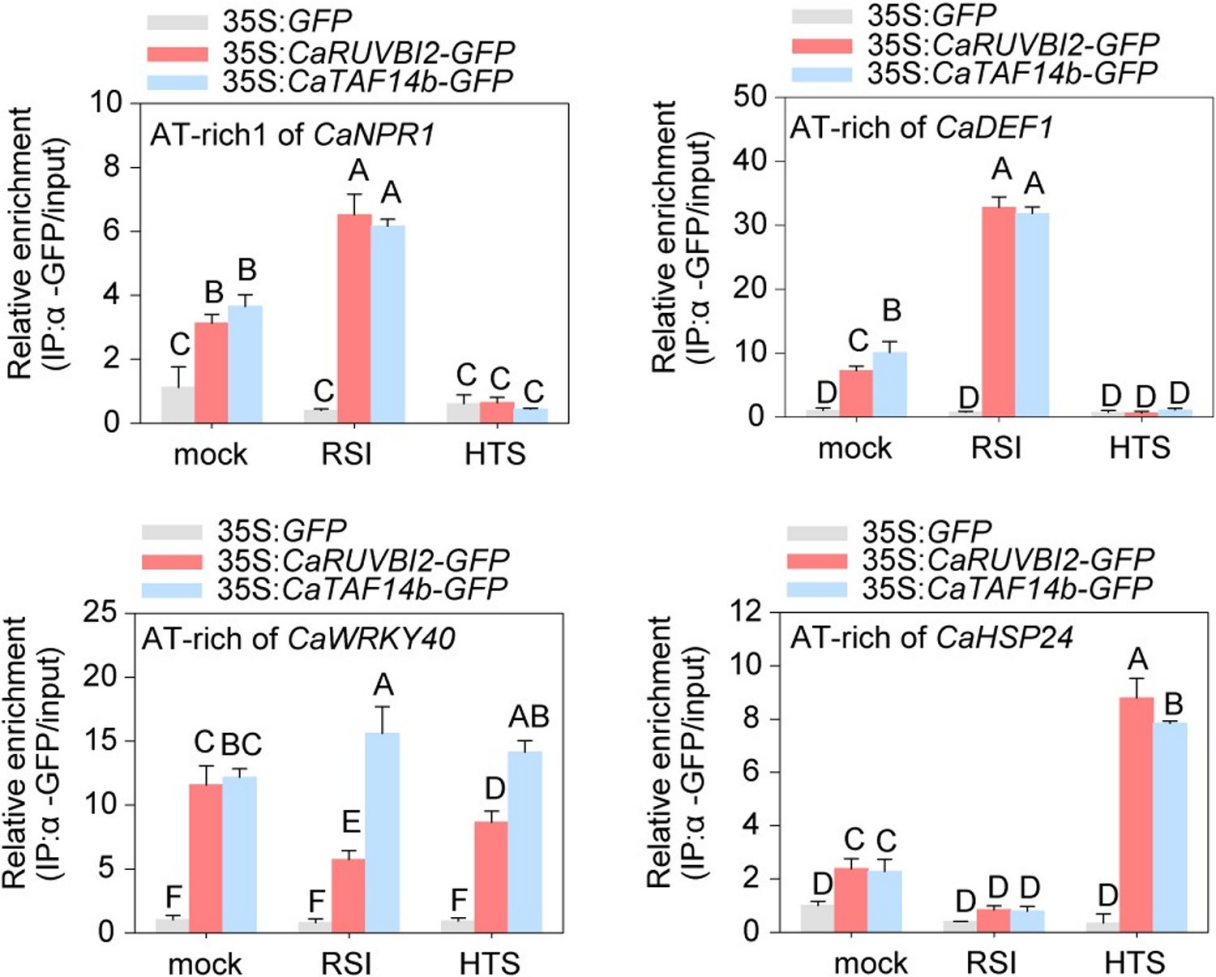
The binding of CaTAF14b and CaRUVBl2 to AT-rich-motifs around the promoters of *CaNPR1*, *CaDEF1*, *CaWRKY40*, and *CaHSP24* is affected by RSI and HTS. Relative enrichment of AT-rich regions within the *CaWRKY40*, *CaNPR1* and *CaDEF1* promoters upon RSI and HTS in the leaves of control plants or plants transiently overexpressing *CaTAF14b/CaRUVBL2*, as determined by ChIP-qPCR.

Finally, to determine whether the specific CaSWC4-mediated deposition of H2A.Z, H3K9ac, and H4K5ac during plant responses to RSI or HTS is dependent on CaTAF14b and CaRUVBL2, we assayed the enrichment of CaTAF14b or CaRUVBL2 on AT rich regions of *CaNPR1*, *CaDEF1*, *CaHSP24*, and *CaWRKY40* DNA under the two conditions. CaSCW4 exhibited non-specific enrichment on AT-rich regions of DNA during plant responses to either RSI or HTS. However, higher levels of CaTAF14b and CaRUVBL2 enrichment were found on the AT-rich regions of *CaNPR1* and *CaDEF1*, but not *CaHSP24*, upon RSI. Meanwhile, when pepper plants were challenged with HTS, higher levels of CaTAF14b and CaRUVBL2 enrichment were found on AT-rich regions of *CaHSP24*, but not *CaNPR1* or *CaDEF1* (Fig 8). Higher levels of CaTAF14b and CaRUVBL2 enrichment were found only on the AT-rich regions of *CaWRKY40* during plant responses to RSI or HTS. Together, these results indicate that CaSWC4 specifically mediates the deposition of H2A.Z, H3K9ac, and H4K5ac around the promoters of its target genes by differentially recruiting CaTAF14b and CaRUVBL2.

## Discussion

Plant responses to biotic and abiotic stresses are largely regulated at the transcriptional level by various TFs, which might function as convergent nodes in the crosstalk between plant responses to different stresses [85]. However, how plants employ a common set of TFs to balance different biological processes remains poorly understood. Here we provide evidence that CabZIP63 and CaWRKY40, two positive regulators of immunity against RSI and thermotolerance in pepper, are recruited to specific chromatin loci by CaSWC4 in a context-dependent manner through interaction with CaTAF14b and CaRUVBL2 to precisely orchestrate multiple chromatin remodeling events, thus differentially regulating the expression of their target genes.

### CaSWC4, CaTAF14b, and CaRUVBL2 positively and coordinately regulate plant responses to HTS and RSI

*CaSWC4*, *CaTAF14b*, and *CaRUVBL2* were all upregulated at the transcriptional level in response to challenges by RSI and HTS in pepper plants. The individual silencing of these genes significantly increased the plants’ susceptibility to RSI and reduced their thermotolerance (Figs 1, S3 and S8). By contrast, the ectopic overexpression of these genes significantly reduced the susceptibility of *N*. *benthamiana* to RSI and enhanced its thermotolerance (Figs 6 and S4). These results are supported by the finding that the immunity-related genes *CaNPR1* and *CaDEF1* and the thermotolerance-related gene *CaHSP24* were induced by transient overexpression of *CaSWC4*, *CaTAF14b*, and *CaRUVBL2* in pepper plants. Moreover, their orthologs were induced by ectopic overexpression of *CaTAF14b* and *CaRUVBL2* in transgenic *N*. *benthamiana* plants with or without stress treatment but were downregulated by the silencing of *CaSWC4*, *CaTAF14b*, or *CaRUVBL2* upon stress treatment (Figs 1, 6, S3 and S10). Together these findings indicate that CaSWC4, CaTAF14b, and CaRUVBL2 are positive regulators of the resistance of pepper to RSI and thermotolerance.

The role of CaSWC4 in immunity against RSI in pepper is consistent to the finding that SWR1 in Arabidopsis regulates plant immunity [51]. This is the first report showing that SWC4, TAF14b, and RUVBL2 function in thermotolerance and that TAF14b and RUVBL2 positively regulate plant immunity against RSI. Similarly, we previously demonstrated that CabZIP63, CaWRKY6, CaWRKY40, and CaCDPK15 are positive regulators of plant responses to RSI and HTS, supporting the notion that plant immunity and thermotolerance are closely related and share a subset of overlapping signaling components. In addition, immunity and thermotolerance are closely related at the epigenetic level, as SWC4, CaTAF14b, and CaRUVBL2 all are members of the chromatin-remodeling complexes SWR1, NuA4, or NuA3 [52, 53]. Perhaps plants employ the same regulatory proteins for different and closely related defense responses to help them rapidly switch from one defense response to another without de novo protein biosynthesis.

### CaSWC4 recruits CabZIP63 and CaWRKY40 to the chromatin in their target loci by orchestrating multiple chromatin remodeling events

It is well established that plant responses to biotic and abiotic stresses are largely regulated by various TFs via massive transcriptional reprogramming. The successful regulation of these target genes, which are distributed in different DNA regions that are wrapped around histones and compacted into chromatin, is dependent on their accessibility to the transcriptional machinery, including TFs. How these chromatin regions are reshaped to become accessible in plants challenged by stress, and how TFs are recruited and target the correct chromatin loci, remain unclear.

Here, we demonstrated that CaSWC4 is activated in pepper in response to HTS and to RSI (S1C Fig). The activated CaSWC4 acts as a positive regulator of plant immunity against RSI and thermotolerance (at least in part) by activating chromatin. Given that subunits such as SWC4 recruit the SWR1 complex to modulate chromatin remodeling, and SWC4 is shared by SWR1 and NuA4 [84], following activation by RSI or HTS, CaSWC4 might recruit not only SWR1 but also the NuA4 complex. As SWC4 interacts with CaTAF14b, a member of the NuA4 and NuA3 complexes (Fig 5), this implies that NuA4 and NuA3 might be indirectly recruited by CaSWC4 via interaction with CaTAF14b. The recruited NuA3 and NuA4 might then promote the biosynthesis of H3K9ac and H4K5ac via acetylation of H3 [53] and H4 [86, 87], respectively (Figs 7 and S10). The activated CaSWC4 also promotes the deposition of H3K9ac, H4K5ac, and H2A.Z at the chromatin loci of immunity- or thermotolerance-related genes and activates their chromatin opening (Fig 4E).

CaSWC4 interacts with H3K9ac, H4K5ac, and H2A.Z and promotes their deposition on the promoters of immunity- or thermotolerance-related genes. This finding is consistent the finding that SWC4 binds to AT-rich DNA elements in Arabidopsis [51] (Fig 4). It is worth pointing out that the interaction of CaSWC4 with H3K9ac, H4K5ac, and H2A.Z was only based on the data from CoIP, we speculate that CaSWC4 might interact indirectly but not directly with histones. The observation that H2A.Z is deposited at the promoters of immunity-related genes by CaSWC4 is consistent with the finding that SWC4 recruits SWR1 to modulate H2A.Z deposition in Arabidopsis [51]. Notably, H2A.Z is generally present in the regulatory regions of specific loci and is believed to generate chromatin regions with particular structural characteristics that either favor rapid transcriptional activation [88] or are associated with transcriptional silencing [89–92]. Here, we found that the deposition of H2A.Z at target loci might be associated with transcriptional activation, since high H2A.Z levels were consistently related to high transcript levels of target genes (Fig 4E). We speculate that CaSWC4 obtains at least some of the energy required for the above process by interacting with CaRUVBL2, a subunit of SWR1 that functions as a molecular motor and has been implicated in chromatin remodeling, to obtain energy from ATP [56]. Our data also indicate that CabZIP63 and CaWRKY40, two TFs that positively regulate plant immunity against RSI and HTS [27, 93], are recruited by CaSWC4 through physical interactions (Fig 2). Thus, CabZIP63 and CaWRKY40 might form a transcription initiation complex to target immunity- or thermotolerance-related genes by binding to their TATA-boxes and RNA polymerase through TFIID [94].

### The immunity-thermotolerance tradeoff relies on the simultaneous, context-specific deposition of CaSWC4, CaTAF14b, and CaRUVBL2 at the promoters of the corresponding genes

Due to the distinct nature of high temperature stress and pathogen attack, plants must adopt completely different defense responses to deal with these two stresses. However, how plants coordinate the recruitment of TFs, recognize their target genes, and perform chromatin remodeling to achieve selective regulation of different target genes by the same TFs remains unclear. Our data indicate that, among the immunity- and thermotolerance-related marker genes examined, the immunity-related genes *CaNPR1* and *CaDEF1* were specifically activated in pepper upon RSI, the thermotolerance-related gene *CaHSP24* was specifically activated in plants challenged with HTS, and *CaWRKY40* was activated under both conditions. The transcriptional activation of these genes was related to the simultaneous deposition of SWC4, CaTAF14b, and CaRUVBL2 at the chromatin in these loci, while the failed transcriptional activation of *CaHSP24* upon RSI and *CaNPR1* or *CaDEF1* upon HTS might be due to the failure of CaTAF14b and CaRUVBL2 to accumulate with CaSWC4 at the promoters of these genes (Figs 7, 8 and S10). This notion is supported by the finding that silencing either *CaTAF14b* or *CaRUVBL2* significantly reduced the enrichment of active chromatin marks H3K9ac, H4K5ac, and H2A.z on the promoters of either *CaNPR1*, *CaDEF1*, or *CaHSP24* and that CaSWC4 failed to regulate plant immunity or thermotolerance in the absence of either CaTAF14b or CaRUVBL2 (S9 Fig). This varied recruitment of CaTAF14b and CaRUVBL2 to the promoter of a given gene might be attributed to unidentified signaling components that are activated by the stress the plant encounters, given that the transient overexpression of *SWC4* upregulated *CaNPR1*, *CaDEF1*, and *CaHSP24* in non-stressed pepper plants (S3 Fig).

Collectively, our findings suggest that CaSWC4, as well as CaTAF14b and CaRUVBL2, are positive regulators of plant responses to RSI and HTS. CabZIP63 and CaWRKY40 are recruited and delivered to their target loci through physical interaction with CaSWC4, which also activate the recruitment or deposition of H2A.Z, H3H9ac, H4K5ac, CaTAF14b, and CaRUVBL2 by physical interaction or binding to the AT-rich elements in the promoters of their target genes. The rapid and accurate transcriptional activation of plant immunity and thermotolerance-related genes depends on the simultaneous and context-specific recruitment of SWC4, TAF14b, and RUVBL2 to their promoters. Therefore, pepper employs common components such as TFs and chromatin-remodeling-related genes to respond to different but closely related stress conditions, allowing it to rapidly and accurately balance stress responses in an energy-efficient manner to enhance survival.

## Materials and methods

### Plant materials and growth conditions

The *Capsicum annuum* inbred line HN42 and *N*. *benthamiana* were used in this study. The seeds were sown in soil. The seedling trays were covered with a transparent plastic dome for 7 d to increase humidity. The seedings were transplanted to a compost soil mix (peatmoss, vermiculite, and perlite, 5:4:1 by volume). The plants were grown in a growth chamber at 28°C under white fluorescent lights at 70–80 mmol photons m^-2^s^-1^, a relative humidity of 70%, and a 16-h light/8-h dark cycle.

### Virus-induced gene silencing

To generate *CaSWC4*, *CaTAF14b*, and *CaRUVBL2* knockdown pepper plants, the VIGS system derived from tobacco rattle virus was used. A specific fragment of the open reading frame and untranslated region of *CaSWC4* were used to construct the vectors *pTRV2-CaSWC4-cds* and *pTRV2-CaSWC4-utr*, respectively. A specific fragment in the 3’UTR of *CaTAF14b* and *CaRUVBL2* was used to construct the vector *pTRV2-CaTAF14b* and *pTRV2-CaRUVBL2*, respectively. VIGS was performed following the method of our previous study [93, 95], the vector *pTRV1*, *pTRV2*:*00*, *pTRV2*:*CaPDS*, *pTRV2*: *CaSWC4-cds*, *pTRV2*: *CaSWC4-utr*, *pTRV2*:*TAF14b* or *pTRV2*:*CaRUVBL2* was transformed into *Agrobacterium* strain GV3101, which were grown in LB medium overnight before collection by centrifugation and resuspension into *Agrobacterium* infiltration buffer to an adjusted OD_600_ = 0.8. Resuspended GV3101 cells carrying the pTRV1 vector were mixed in a 1:1 ratio with resuspended GV3101 cells bearing the pTRV2:*00*, pTRV2:*CaPDS*, *pTRV2*: *CaSWC4-cds*, *pTRV2*: *CaSWC4-utr*, *pTRV2*: *TAF14b* or *pTRV2*:*CaRUVBL2*, the mixture was incubated for 3 h on a shaker set at 60 rpm, and then infiltrated into the cotyledons of pepper seedlings. The gene silencing procedure was monitored by occurrence of bleaching in plants infiltrated with pTRV2:*CaPDS*.

### Generation of transgenic *N*. *benthamiana* plants

Agrobacterium-mediated transformation of *N*. *benthamiana* was conducted using the leaf disc method [96], following the method of our previous study [97]. *Agrobacterium tumefaciens* containing *35S*:*CaSWC4-YFP*, *35S*:*CaTAF14b-GFP*, or *35S*:*CaRUVBL2-GFP* was used to transform leaf discs of *N*. *benthamiana*. Independent T_0_ transgenic *N*. *benthamiana* lines were selected on10mM basta (glufosinate, Sigma, 45520) and later confirmed by PCR with specific primers (S1 Table). T_0_ plants were allowed to self-pollinate and seeds of each transgenic plants were harvested individually. In similar way, we got the plants of T_2_ and T_3_ lines, and all subsequent plant work was performed on homozygous T_3_ lines.

### Inoculation of plants with RSI and treatment with HTS

The plants were inoculated with *R*. *solanacearum* strain FJC100301 [27] through root irrigation or by leaf injection, following the method of our previous study [97]. For root irrigation, 1 mL of *R*. *solanacearum* suspension diluted to 10^8^cfu ml^-1^ (OD_600_ = 0.8)with deionized water was irrigated to each pot containing one plant, whose roots was slightly mechanical damaged; for leaf inoculation, about 100 uL of *R*. *solanacearum* suspension diluted to 10^3^ cfu ml^-1^ (OD_600_ = 0.3) was injected to each site in the leaf, the plants or leaves treated with deionized water were used as controls. For HTS treatment, the plants were placed to a condition of 42°C and 90% humidity in a light incubator, the phenotype of the plants was monitored. The plants or leaves were harvested at indicated time for measuring the growth of *R*. *solanacearum* (CFU), RNA extraction, and so on.

### Agrobacterium-mediated transient expression and subcellular localization

GV3101 cells containing *35S*:*CaSWC4-YFP/-MYC*, *35S*:*CaTAF14b-GFP*, *35S*:*CaRUVBL2-GFP*, *35S*:*YFP* or *35S*:*GFP* were used for transient expression analysis in pepper and subcellular localization assays in epidermal cells of leaves of *N*. *benthamiana* plants following the method described previously [97]. About 100 uL of suspension GV3101 containing different vectors diluted to 10^8^cfu ml^-1^ (OD_600_ = 0.8) with induction medium (10 mM MES, 10 mM MgCl_2_, 200 μM acetosyringone, pH 5.2) was infiltrated into leaves of *N*. *benthamiana* with injection without a needle, and the fluorescent signal from Agrobacterium-infiltrated *N*. *benthamiana* leaves was observed at 48 hpi. YFP fluorescence was collected on a confocal microscope (TCS SP8, Leica Microsystems, Germany).

### Histochemical staining and conductivity leakage measurements

Trypan blue and DAB staining were used to assess hypersensitive response (HR)-like cell death and H_2_O_2_ accumulation in pepper leaves, respectively, as described previously [95]. Ion leakage from leaves was analyzed by measuring conductivity as described previously [98].

### Pull-down and microscale thermophoresis assays

The pull-down assay was performed following the method of our previous study [99], briefly, the indicated proteins were fused with 6×His or GST tags and expressed in *Escherichia coli* strain BL21. The supernatant containing 6×His- and GST-tagged soluble proteins was mixed and incubated with BeaverBeads IDA-Nickel (Beaver Biosciences, China) at 4°C for 3 h. The beads were collected and washed four times with Tris buffer. The eluted proteins were separated by SDS-PAGE and detected using anti-His and anti-GST antibodies.

MST was performed as previously described [99, 100]. CaWRKY40-GFP, CabZIP63-GFP, CaTAF14b-GFP, CaRUVBL2-GFP fusion protein, or GFP (as a control) was used for the fluorescent label and CaSWC4-6×His fusion protein was used for the non-fluorescent label as described by Huang *et al*. [98]. CaSWC4-6×His fusion protein was diluted to a range of concentrations from 1.0E^–10^ mM to 1.0E^–3^ mM, then incubated with 20 mM of the labeled protein for 10 min in interaction buffer. The samples were then loaded into Monolith NT.115 Capillaries (Cat#MO-K002, NanoTemper Technologies, Germany) using 50% IR laser power and an LED excitation source, where λ = 470 nm at room temperature. We used the Nano Temper Analysis 1.2.20 software to fit the data and determine apparent K_d_ values [101, 102].

### BiFC and Co-IP assays

The BiFC and Co-IP assays to detect protein interactions were performed in leaves of *N*. *benthamiana* or pepper plants using *A*. *tumefaciens*-mediated transient expression. For the BiFC assay, the indicated protein was fused with the N- or C-terminal part of YFP and coexpressed in *N*. *benthamiana* leaves throuth agro-infiltration. At 48 hpi, YFP signals were imaged under a confocal microscope (Leica, Solms, Germany).

For the Co-IP assay, the indicated gene was fused with GFP- or MYC tag and co-expressed in leaves of pepper via agroinfiltration. At 48 hpi, the total proteins were extracted from the agro-infiltrated pepper leaves using protein extraction buffer [25 mM Tris-HCl, pH 7.5, 10 mM NaCl, 0.1% Triton X-100, 5 mM DTT, 1× Complete protease inhibitor cocktail (Sigma-Aldrich, St. Louis, MO, USA)]. The extracted proteins were incubated with anti-MYC or anti-GFP magnetic beads (Sigma-Aldrich) at 4°C overnight. The beads were then collected with a magnet and washed three times with protein extraction buffer. Eluted proteins were immunoblotted using anti-GFP or anti-MYC antibody (Abcam, Cambridge, UK). To examine the binding of recombinant CaSWC4 to modified histone, the extracted proteins were incubated with anti-GFP magnetic beads (Sigma-Aldrich) at 4°C overnight. Eluted proteins were detected using modified histone antibodies (Abcam, Cambridge, UK).

### Chromatin immunoprecipitation (ChIP) and ChIP-seq

ChIP assay was performed according to the protocol of Shen *et al*. [93].The leaves of pepper plants(or VIGS pepper plants) transiently expressing the indicated proteins by agroinfilatration were crosslinked with 1% formaldehyde, and then were sheared into fragments of 300–500 bps by sonication and immunoprecipitated with anti-GFP antibody (Abcam, Cambridge, UK). In ChIP assay for measuring the enrichments of histone modification markers on specific chromatin loci, the leaves were not crosslinked, and the chromatin was extracted from the agroinfiltrated leaves and sheared into fragments of 300–500 bps by sonication. The DNA mixture was immunoprecipitated with anti-H3, anti-H2A.Z, anti-H3k9ac, anti-H3k9me2 or anti-H4k5ac (Abcam, Cambridge, UK). The acquired DNAs by immunoprecipitation with different antibodies were purified and used as template for ChIP**-**qPCR using the specific primer pairs for ChIP**-**qPCR (S1 Table).

For ChIP-seq, we infiltrated 30 fully expanded leaves of pepper plants at the 6-leaf stage with GV3101 cells harboring the binary vector *35S*:*CaWRKY40-GFP*. At 48 hpi, the infiltrated leaves were harvested and cross-linked with 1% formaldehyde, and the chromatin was isolated and subjected to ChIP following the above-mentioned method. The decross-linked and purified DNA sample was subjected to linear DNA amplification (LinDA) in order to generate sufficient material to construct ChIP-seq sequencing libraries using a NEBNext ChIP-seq Library Pre Reagent Set for Illumina (New England Biolabs, Ipswich, MA). DNA sequencing was performed on an Illumina Hiseq2500 platform (Novogene, Beijing, China) and resulted in about 10 million 100 bp single-end reads per sample. We removed low quality reads, those with over 15% ambiguous bases, reads contaminated with 5’ barcode, and reads without 3’ linker sequences or inserts, trimmed the 3’ linker sequences, and discarded reads shorter than 18 nt after data cleaning. The remaining reads were aligned to the pepper reference genome by BWA (Burrows Wheeler Aligner) [103]. DNA fragment sizes were predicted using MACS2 software, which we then used for subsequent peak analysis. We also used the MACS2 software (with threshold *q*-value = 0.05) to detect signal peaks, as well as for analysis of the number, width, and distribution of peaks, and the underlying genes identified by the peaks [104].

### RNA extraction and RT-qPCR assay

Total RNA was extracted from plant materials using TRIzol (Invitrogen, Canada) reagent. Approximately 1 μg of total RNA was used to generate cDNA using reverse transcriptase (TaKaRa Biotechnology, Japan). To measure gene expression, RT-qPCR assay was performed using SYBR Premix Ex Taq (Perfect Real Time; TaKaRa) using specific primer pairs (S1 Table). The expression levels of the target genes were normalized to that of the internal control gene *CaActin* (GQ339766). Each assay was performed using four independent biological repeats. Data were analyzed by the Livak method [105] and expressed as the normalized relative expression level (2^-ΔΔCT^) of the respective gene. Statistical significance was evaluated by Student’s *t* test (significance, *P* < 0.05 or *P* < 0.01).

### RNA-seq analysis

For RNA-seq assay, total RNA was extracted from the roots of pepper line HN42 at the ten-leaf stage challenged with RSI or HTS (using the plants that were placed under the condition of room temperature as mock treatment), and the plants were harvested at 48 hpi(t). We also collected roots from *CaWRKY40*-silenced and wild-type pepper plants, total RNA was extracted with the MagMAX-96 Total RNA Isolation Kit (Ambion, AM1830) according to the manufacturer’s instruction. mRNA sequencing library was constructed with barcodes using the TruSeq RNA Sample Preparation Kit (Illumina) with three biological replicates per treatment, and RNA-seq was performed on Illumina HiSeq2500 platform by Novogene (Beijing, China) as 125 bp/150 bp paired-end reads.

We downloaded the reference genome and gene model annotation files from the pepper genome database of Zunla line(http://peppersequence.genomics.cn), and generated genome indices and aligned the cleaned paired-end reads to the reference genome using HISAT2. The read counts per gene was obtained with HTSeq v0.6.1 [106], which were then converted to fragments per kilobase of transcript per million mapped reads (FPKM) by normalizing read counts to gene length. We determined differential expression between two conditions/groups (three biological replicates per treatment) using the EdgeR package. The resulting *P* values were adjusted using the Benjamini and Hochberg approach for controlling the false discovery rate [107]. The genes in data of RNA-seq in the present study were functionally annotated based on gene function annotation in Zunla genome database, some interesting genes were picked out to draw heatmap of gene expression.

### Electrophoretic mobility shift assay

CaSWC4-6×His was expressed in *E*. *coli* strain BL21 and purified using Ni-nitrilotriacetic acid resin (Qiagen). The wild-type or mutated AT-rich fragment was synthesized by PCR using a single-stranded primer and another single-stranded primer labeled with Cy5.

EMSA was carried out as described previously [108]. The recombinant CaSWC4-6×His proteins were incubated with wild-type or mutated probe, which was labeled with the Cy5 fluorochrome, and 5× binding buffer (1 M Tris-HCl, pH 7.5, 5 M NaCl, 1 M KCl, 1 M MgCl_2_, 0.5 M EDTA, pH 8.0, 10 mg/ml bovine serum albumin). The mixture was separated by PAGE and scanned on the Odyssey CLX imaging system (LI-COR).

### Chlorophyll fluorescence spectrophotometry

We used a MINI Imaging PAM instrument (Heinz Walz GmbH, Effeltrich, Germany) to measure F_v_/F_m_ and △F/F_m_′ values from pepper and *N*. *benthamiana* leaves. The plants were adapted to darkness for 15 min before being placed into the instrument for measurements according to the method of Schreiber [109].

## Supporting information

S1 Table Primers used in this study. (DOCX)

S2 Table Disease index for *Ralstonia solanacearum*-infected pepper plants. (DOCX)

S3 Table The result of WRKY40 ChIP-seq and RNA-seq. (XLSX)

S1 Fig *CaSWC4* is upregulated by *R*. *solanacearum* or HTS and by transient overexpression of *CaWRKY40*. **A.** The enrichment of CaWRKY40 on CaSWC4 promoter determined by ChIP-seq was checked using IgV, and 100–200 bp primers based on the corresponding enrichment region. B. FPKM (Fragments per Kilobase Million) of SWR1-C in pepper plant silencing with *CaWRKY40* based the RNA-seq data set. C. Enrichment of CaWRKY40 at the W-box-containing promoter fragment of *CaSWC4* (P^Wb^) in pepper plants challenged with RSI or HTS. D. *CaSWC4* was upregulated by transient overexpression of *CaWRKY40* in leaves of pepper plants challenged with RSI or HTS. In C and D, Data are shown as means ± standard error of three replicates, asterisks above the bars indicated significant differences among means (*P* < 0.01), as calculated with t-test. E. Relative expression levels of *CaSWC4* in roots of pepper plants challenged with RSI or HTS at 1, 3, 6, 12, 24, and 48 hpt (i). Error bars show standard error; data represent the means ± SD obtained from four replicates. Different capital letters above the bars indicate significant differences among means (*P* < 0.01), as determined by Fisher’s protected least-significant-difference (LSD) test.
(TIF)

S2 Fig Nuclear localization of CaSWC4 in *N*. *benthamiana* epidermal cells. Leaves of *N*. *benthamiana* plants were infiltrated with GV3101 cells containing *35Spro*:*CaSWC4-YFP* (using *35Spro*:*YFP* as a control). Subcellular localization of the CaSWC4-YFP fusion protein or control YFP was observed under a fluorescent confocal microscope at 24 hpi. Fluorescence images (left), bright-field images (middle), and the corresponding overlay images (right) of representative cells expressing YFP or CaSWC4-YFP fusion protein are shown. Bars = 50 μm.
(TIF)

S3 Fig Transient overexpression of *CaSWC4* induces HR-like cell death and the expression of immunity- and thermotolerance-related marker genes. **A and B,** confirmation of the transient overexpression of *CaSWC4* by RT-qPCR and immunoblot analysis with anti-GFP antibody. **C.** Transient overexpression of *CaSWC4* induced higher levels of ion leakage (displayed as relative conductivity) in pepper leaves. **D.** Fv/Fm shown in pseudo-color images in CaSWC4 overexpressing pepper leaves challenged with HTS compared to that in the mock-treatment. The lower half leaf blades were kept in room temperature, while the upper half leaf blades were inserted into water at 42°C for 2 min. The pseudo-color image was detected immediately after HTS treatment. **E.** △F/Fm’ in leaves of CaSWC4 overexpressing pepper leaves challenged with HTS. **F.** Transient overexpression of *CaSWC4* induced clear HR mimic cell death (as revealed by darker trypan blue staining) and H_2_O_2_ accumulation (as revealed by darker diaminobenzidine [DAB] staining). **G.** *CaNPR1*, *CaDEF1*, and *CaHSP24* were upregulated by transient overexpression of *CaSWC4* in the leaves of pepper plants. In **B, E, F** and **G,** data are shown as means ± standard error of three replicates, asterisks above the bars indicated significant differences among means (*P* < 0.01), as calculated with t-test.
(TIF)

S4 Fig The effect of heterologous overexpression of *CaSWC4* on the response of *N*. *benthamiana* plants to RSI and HTS. **A.** Confirmation of *CaSWC4* overexpression by RT-qPCR and immunoblot analysis with anti-GFP antibody in the two T_2_ transgenic *N*. *benthamiana* lines. **B** and **C,** *N*. *benthamiana* plants overexpressing *CaSWC4* displayed higher levels of resistance to *R*. *solanacearum* infection (B) and HTS (C) than wild-type control plants. In **A**, data are shown as means ± standard error of four replicates; different capital letters indicate significant differences among means (*P* < 0.01), as determined by Fisher’s protected LSD test.
(TIF)

S5 Fig The roles of CaWRKY40/CabZIP63 in immunity and thermotolerance in pepper are dependent on CaSWC4. **A.** Confirmation of transient overexpression of *CaWRKY40-GFP* or *CabZIP63-GFP* in the leaves of *CaSWC4* silenced (TRV:*CaSWC4*) pepper plants by qRT-PCR. **B.** CaWRKY40-GFP and CabZIP63-GFP levels in the leaves of *CaSWC4*-silenced pepper plants transiently overexpressing these proteins, as measured by immunoblot analysis with anti-GFP antibody. **C.** HR cell death induced by transient overexpression of *CaWRKY40* or *CabZIP63* was significantly blocked by the silencing of *CaSWC4*. **D.** Upregulation of immunity-related genes *CaNPR1* and *CaDEF1* triggered by the transient overexpression of *CaWRKY40* or *CabZIP63* was blocked by the silencing of *CaSWC4*. In **A** and **D**, data are shown as means ± standard error of four replicates; different capital letters indicate significant differences among means (*P* < 0.01), as determined by Fisher’s protected LSD test.
(TIF)

S6 Fig Binding of CaSWC4-6×his to AT-rich-elements, as determined by EMSA. EMSA to examine the binding of CaSWC4-6×His to the *CaPR1*, *CaNPR1*, *CaDEF1*, and *CaHSP24* promoters containing AT-rich-elements and to promoter fragments of *CaPR1*, *CaNPR1*, *CaDEF1*, and *CaHSP24* containing mutated AT-rich-elements.
(TIF)

S7 Fig The binding of CaSWC4 to AT-rich-motifs around the *CaNPR1*, *CaDEF1*, *CaWRKY40*, and *CaHSP24* promoters is unaffected by RSI and HTS. Relative enrichment of AT-rich motifs within the promoters of *CaWRKY40*, *CaNPR1*, and *CaDEF1* upon RSI and HTS treatment in control pepper leaves or leaves transiently overexpressing *CaSWC4-YFP*, as determined by ChIP-qPCR.
(TIF)

S8 Fig Effect of *CaRUVBL2/CaTAF14b* silencing on the resistance of pepper to RSI and HTS. **A.** Relative transcript levels of *CaRUVBL2*, *CaTAF14b*, and *CaWRKY40* in *CaRUVBL2/CaTAF14b*-silenced pepper plants challenged with RSI or HTS. **B.** Effect of *CaRUVBL2/CaTAF14b* silencing on plant resistance to RSI or HTS. H_2_O_2_ and ROS accumulation, as observed by DAB and NBT staining of the leaves of *CaRUVBL2-* or *CaTAF14b*-silenced pepper plants challenged with HTS. In A, data are shown as means ± standard error of four replicates; different capital letters indicate significant differences among means (*P* < 0.01), as determined by Fisher’s protected LSD test.
(TIF)

S9 Fig CaSWC4 and CaTAF14b/CaRUVBL2 cooperatively regulate immunity and thermotolerance in pepper. **A.** The silencing of *CaTAF14b* or *CaRUVBL2* significantly blocked the accumulation of CaSWC4-MYC and HR cell death in the leaves of pepper plants transiently overexpressing *CaSWC4-MYC*, as revealed by immunoblotting with anti-MYC antibody. **B.** *CaTAF14b/CaRUVBL2* silencing significantly blocked ion leakage (displayed as relative conductivity) induced by transient overexpression of *CaSWC4* in the leaves of pepper plants. In B, data are shown as means ± standard error of four replicates; different capital letters indicate significant differences among means (*P* < 0.01), as determined by Fisher’s protected LSD test.
(TIF)

S10 Fig The silencing of *CaRUVBL2* or *CaTAF14b* significantly blocks the deposition of H2A.Z, H3K9me2, and H4K5Ac on the TSSs and promoters of immunity- and thermotolerance-related marker genes. A. *CaRUVBL2* and *CaTAF14b* were successfully silenced by VIGS, and *CaSWC4* was successfully transiently overexpressed in both *CaRUVBL2-* and *CaTAF14b*-silenced pepper leaves challenged by RSI or HTS. B. The enrichment of H2A.Z, H3K9Ac, and H4K5ac (enrichment of histone 3 as internal) at the TSSs of *CaNPR1*, *CaDEF1*, and *CaHSP24* was enhanced by transient overexpression of *CaSWC4*, but this upregulation was blocked by silencing of *CaRUVBL2* or *CaTAF14b*, while the enrichment of H3K9me2 at the TSSs of the tested marker genes was reduced by transient overexpression of *CaSWC4* but was not affected by the silencing of *CaRUVBL2* or *CaTAF14b*. C. Immunity-related *CaNPR1* and *CaDEF1* were upregulated by *CaSWC4* transient overexpression in leaves of pepper plants challenged by RSI, and thermotolerance-related *CaHSP24* was upregulated by transient overexpression of *CaSWC4* in the leaves of pepper plants challenged by HTS, but not when *CaRUVBL2* or *CaTAF14b* was silenced. Data are shown as means ± standard error of three replicates, asterisks above the bars indicated significant differences among means (*P* < 0.01), as calculated with t-test. In A and C, data are shown as means ± standard error of four replicates; different capital letters indicate significant differences among means (*P* < 0.01), as determined by Fisher’s protected LSD test.
(TIF)
