# Supplementary material for: CaSWC4 regulates the immunity-thermotolerance tradeoff by recruiting CabZIP63/CaWRKY40 to target genes and activating chromatin in pepper
Source: PLoS Genet. 2022 Feb 28;18(2):e1010023. doi: 10.1371/journal.pgen.1010023 (PMC8884482; doi:10.1371/journal.pgen.1010023)
Supplement: S1 Table — (DOCX) [file pgen.1010023.s001.docx]

|  | **Gene** | **GenBank**  **Accession #** | **F_primer** | **R_primer** |
| --- | --- | --- | --- | --- |
| **Primers used to study *CaSWC4*** | ***CaSWC4*^1^** | XP_016539729.1 | GGGGACAAGTTTGTACAAAAAAGCAGGCTTCATGGATGCGAAGGACATCTT | GGGGACCACTTTGTACAAGAAAGCTGGGTCTCATCCATCAGTCTTTAACT |
|  | ***CaSWC4*-GFP^2^** | XP_016539729.1 | GGGGACAAGTTTGTACAAAAAAGCAGGCTTCATGGATGCGAAGGACATCTT | GGGGACCACTTTGTACAAGAAAGCTGGGTCTCCATCAGTCTTTAACTTGC |
|  | ***CaSWC4-utr*-VIGS^3^** | XP_016539729.1 | GGGGACAAGTTTGTACAAAAAAGCAGGCTTC ACTTCTTCTGCCATTGTGAT | GGGGACCACTTTGTACAAGAAAGCTGGGTC CCATCCTGGTAAGCAATCTAA |
|  | ***CaSWC4-cds*-VIGS^4^** | XP_016539729.1 | GGGGACAAGTTTGTACAAAAAAGCAGGCTTC AAGCAGGTACAATACAAGGA | GGGGACCACTTTGTACAAGAAAGCTGGGTC ATGGAACTTCTGATGATCGT |
| **Primers used for *CaTAF14b/CaRUVBL2 VIGS*** | ***CaTAF14b*^1^** | XP_016560530.1 | GGGGACAAGTTTGTACAAAAAAGCAGGCTTCGGATGCCTCTCGACTCGCCGGCCGTC | GGGGACCACTTTGTACAAGAAAGCTGGGTCTCAGAACAGAAGATGTTTAC |
|  | ***CaRUVBL2*^1^** | PHT83058.1 | GGGGACAAGTTTGTACAAAAAAGCAGGCTTCGGATGACTATTTATTTCTTGCT | GGGGACCACTTTGTACAAGAAAGCTGGGTCGGACACCATTGCAGTAG |
|  | ***CaTAF14b* -GFP^2^** | XP_016560530.1 | GGGGACAAGTTTGTACAAAAAAGCAGGCTTCGGATGCCTCTCGACTCGCCGGCCGTC | GGGGACCACTTTGTACAAGAAAGCTGGGTCGAACAGAAGATGTTTAC |
|  | ***CaRUVBl2* -GFP^2^** | PHT83058.1 | GGGGACAAGTTTGTACAAAAAAGCAGGCTTCGGATGACTATTTATTTCTTGCT | GGGGACCACTTTGTACAAGAAAGCTGGGTCCACCATTGCAGTAG |
|  | ***CaTAF14b -utr*-VIGS^3^** | XP_016560530.1 | GGGGACAAGTTTGTACAAAAAAGCAGGCTTCGGTGTCCTGGATAACGCAGGAT | GGGGACCACTTTGTACAAGAAAGCTGGGTCTGCCAGAGTTCACAGAGCAG |
|  | ***CaRUVBl2 -utr*-VIGS^3^** | PHT83058.1 | GGGGACAAGTTTGTACAAAAAAGCAGGCTTCGGAGCTTCTCGACTTGGGATTCA | GGGGACCACTTTGTACAAGAAAGCTGGGTCACACAGAAAAATAGAAACTGGTGCA |
| **Primers used for qPCR analysis** | ***CaSWC4*-qPCR** | XP_016539729.1 | TTGAGCGAAAACGTGCACTG | CAGGAGCAGCATCTGACACA |
|  | ***CaTAF14b*-qPCR** | XP_016560530.1 | ACGCAGAGAATGAGCATGGT | AAATGTTGCAGGAAGCCGTG |
|  | ***CaRUVBl2*-qPCR** | PHT83058.1 | ATGGCAATGGGTGAGGTAAAA | GGACACCATTGCAGTAGTTTC |
|  | ***CaWRKY40*-qPCR** | AAX20040.1 | GGTGTGGCAGATGATAGTGC | CCAGGCACAACATCCAAGT |
|  | ***CabZIP63*-qPCR** | XP_016537747.1 | ACGACATTGCCGATCAATTA | GCAAACGATGCGGTATTAGA |
|  | ***CaHSP2*4-qPCR** | HM132040 | GTTCGTCTAGCAGTTTGGTTCGGTTG | GTAATTTAACTAAACAGACTCTTACAACC |
|  | ***CaNPR1*-qPCR** | X61679.1 | ACTTCTTCGCCGACGCCAAG | GCCAACACATTCACCAGAGCATC |
|  | ***CaDEF1*-qPCR** | AF442388 | GTGAGGAAGAAGTTTGAAAGAAAGTAC | TGCACAGCACTATCATTGCATACAATTC |
|  | ***CaACTIN*** | GQ339766 | AGGGATGGGTCAAAAGGATGC | GAGACAACACCGCCTGAATAGC |
| **Primers used for ChIP-PCR and EMSA** | **PNPR1-AT-EMSA** | X61679.1 | Cy5-ATATATACTAAAGGTATAATATAAATCTTTTTATTTTTTT | AAAAAAATAAAAAGATTTATATTATACCTTTAGTATATAT |
|  | **PDEF1-AT-EMSA** | AF442388 | Cy5-TTTCCCAAAAATCGTAATGGAGTCCGACCATAACGAATT | AATTCGTTATGGTCGGACTCCATTACGATTTTTGGGAAA |
|  | **PHSP24-AT-EMSA** | HM132040 | Cy5 TATAAACTAAATAAAAGAGCCCAACCCAAAAAACTAGTTT | AAACTAGTTTTTTGGGTTGGGCTCTTTTATTTAGTTTATA |
|  | **PWRKY40-AT-EMSA** | AAX20040.1 | Cy5 CAAAAAATTCAATCAATTAAATATTCTGCATTTCAAGAAT | ATTCTTGAAATGCAGAATATTTAATTGATTGAATTTTTTG |
|  | **PNPR1-AT1** | X61679.1 | ACAATGAATCTCATGCATGCAT | TGGGTTAGTTATGCTCTTATACAGTGT |
|  | **PNPR1-AT2** | X61679.1 | TGGTCCCCTTCCTTCCTCTT | TTGCTTCCTCTGACTGAGCG |
|  | **PNPR1-AT3** | X61679.1 | TGGATCCAATGCATGCATTAACA | AGTTTCTTTTGCAGTAATATCCTCCA |
|  | **PDEF1-AT** | AF442388 | CGCAGACCATCACTTCGAGT | TGGGTTCTTCCACTTATCAAACT |
|  | **PHSP24-AT** | HM132040 | TCTCGTGTATGTTGGACCCT | CGACGCTTCCGGATTGTTCA |
|  | **PWRKY40-AT** | AAX20040.1 | TGTCACTACTTGTTCATCTCAACA | TAATTGCTCGCCCCTCTGAG |
|  | **PNPR1-Tss** | X61679.1 | CAAAAAAATGGCAAACTCTC | AGGGAAAAGAAGAGGAGATA |
|  | **PDEF1-Tss** | AF442388 | CACGTGACACTCATTTCAGCA | ACAGAGCTTACCTTCGTGCT |
|  | **PHSP24-Tss** | HM132040 | TGAGTTTCTCGAATCCTTTTTCCC | TTCGCCGACTTAGCTTCACG |
|  | **PWRKY40-Tss** | AAX20040.1 | ACAACTTGGTCAGCTTGGTCT | AGCTCTTCTAGTCTTTCTTCAGGTG |
|  | **PNPR1-Wbox** | X61679.1 | GAGCTAATAAATGAGCAGGT | CAATTATCCAGGAAAATGCT |
|  | **PDEF1-Wbox** | AF442388 | GCTATAAAACCATCATATTG | TAGTGTACGTGTAGGACCAT |
|  | **PHSP24-Wbox** | HM132040 | TTTGACTCAATTTACTAGCG | CGTAAACTATTGATATTTTA |
|  | **PWRKY40-Cbox** | AAX20040.1 | TATTCTCAAAAAATTCAATC | ATTCAAGTGTTTGTTTACAA |
|  | **PWRKY40-Gbox** | AAX20040.1 | AACCAAGATTGTACTATAGC | AATTGCCCTTTTAAGAAGAG |
| **Primers used for qPCR analysis** | ***NtNPR1*** | LOC107831756 | GTGGACAACGAGTGCTCTCA | GCTGCAGCTTTGCCAAGAAT |
|  | ***NtAPX*** | XP_016432750.1 | CGCTCCTCTTATGCTCCGTCTT | GGTGGCTCTGTCTTGTCCTCTC |
|  | ***NtDEF1*** | ABU40984.1 | GCCTTACCAAACCACCATGC | GCTGCAGCCAAAGTTTTTGC |
|  | ***NtEF-1a*** | D63396 | TGCTGCTGTAACAAGATGGATGC | GAGATGGGGACAAAGGGGATT |

**S1 Table. Primers used in this study**

^1^Primers used to clone the *CaSWC4/CaTAF14b/CaRUVBl2* full-length CDS

^2^Primers used for *35Spro:CaSWC4/CaTAF14b/CaRUVBl2-GFP* vector construction

^3^Primers used for TRV:*CaSWC4/CaTAF14b/CaRUVBl2* -utr vector construction

^4^Primers used for TRV:*CaSWC4/CaTAF14b/CaRUVBl2-cds* vector construction
