# Supplementary material for: CaSWC4 regulates the immunity-thermotolerance tradeoff by recruiting CabZIP63/CaWRKY40 to target genes and activating chromatin in pepper
Source: PLoS Genet. 2022 Feb 28;18(2):e1010023. doi: 10.1371/journal.pgen.1010023 (PMC8884482; doi:10.1371/journal.pgen.1010023)
Supplement: S2 Table — (DOCX) [file pgen.1010023.s002.docx]

**S2 Table. Criteria for disease grading to calculate the disease index of pepper plants inoculated with *Ralstonia solanacearum***

| **Score** | **Condition** |
| --- | --- |
| 0 | Normal, asymptomatic plants. |
| 1 | Plants with slight withering: the basal 1–2 leaves are withered; the apical region of the plant is normal. |
| 2 | Except for the top leaves, half of all leaves are withered; the apical region of the plant is normal. |
| 3 | 2/3 of the leaves are withered; the top of the plant is normal. |
| 4 | Whole plant has withered or died. |
