# Supplementary figures and images for: CaSWC4 regulates the immunity-thermotolerance tradeoff by recruiting CabZIP63/CaWRKY40 to target genes and activating chromatin in pepper
Source: PLoS Genet. 2022 Feb 28;18(2):e1010023. doi: 10.1371/journal.pgen.1010023 (PMC8884482; doi:10.1371/journal.pgen.1010023)

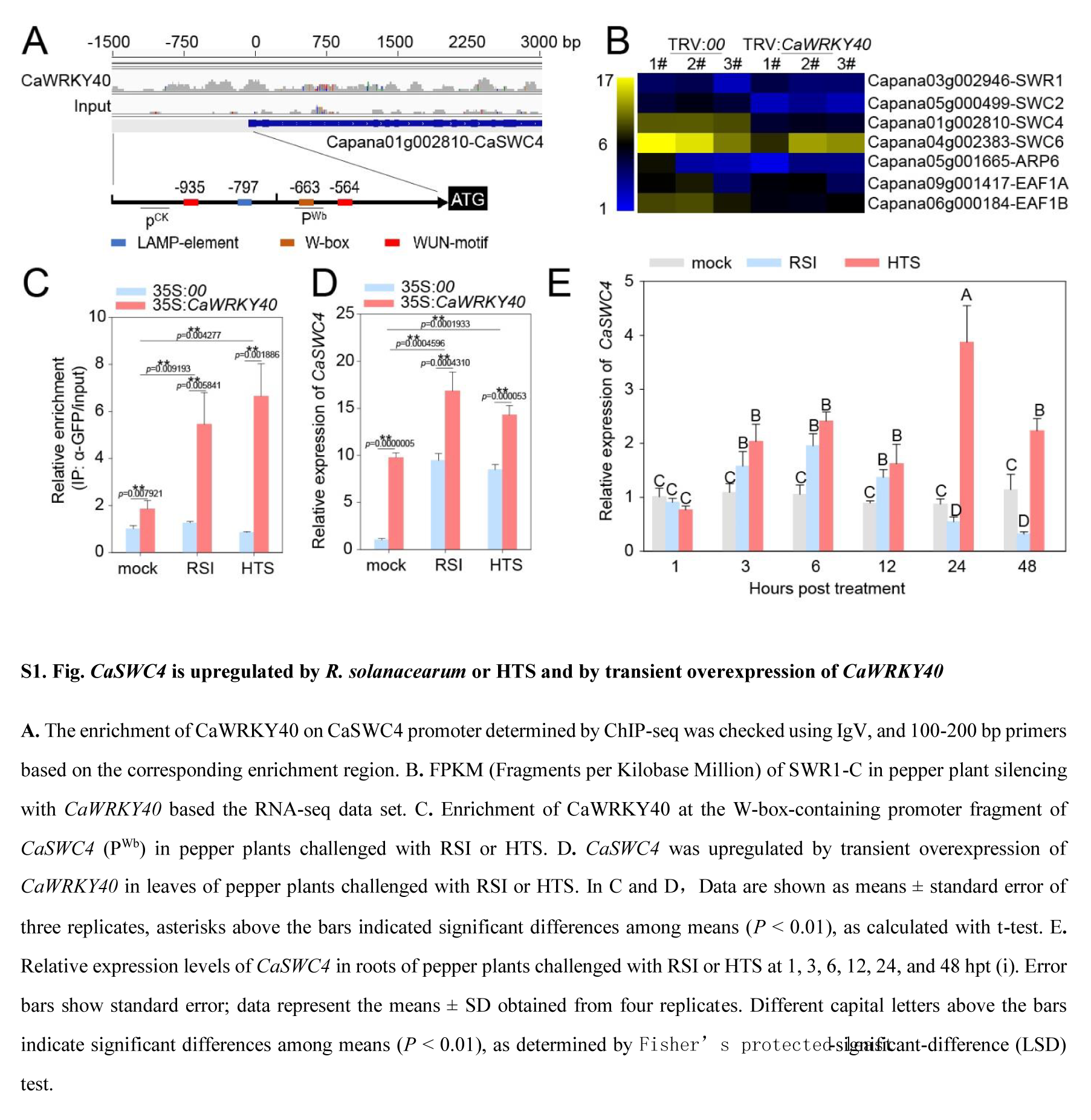

Supplement: S1 Fig — A. The enrichment of CaWRKY40 on CaSWC4 promoter determined by ChIP-seq was checked using IgV, and 100–200 bp primers based on the corresponding enrichment region. B. FPKM (Fragments per Kilobase Million) of SWR1-C in pepper plant silencing with CaWRKY40 based the RNA-seq data set. C. Enrichment of CaWRKY40 at the W-box-containing promoter fragment of CaSWC4 (PWb) in pepper plants challenged with RSI or HTS. D. CaSWC4 was upregulated by transient overexpression of CaWRKY40 in leaves of pepper plants challenged with RSI or HTS. In C and D, Data are shown as means ± standard error of three replicates, asterisks above the bars indicated significant differences among means (P < 0.01), as calculated with t-test. E. Relative expression levels of CaSWC4 in roots of pepper plants challenged with RSI or HTS at 1, 3, 6, 12, 24, and 48 hpt (i). Error bars show standard error; data represent the means ± SD obtained from four replicates. Different capital letters above the bars indicate significant differences among means (P < 0.01), as determined by Fisher’s protected least-significant-difference (LSD) test. (TIF) [file pgen.1010023.s004.tif]

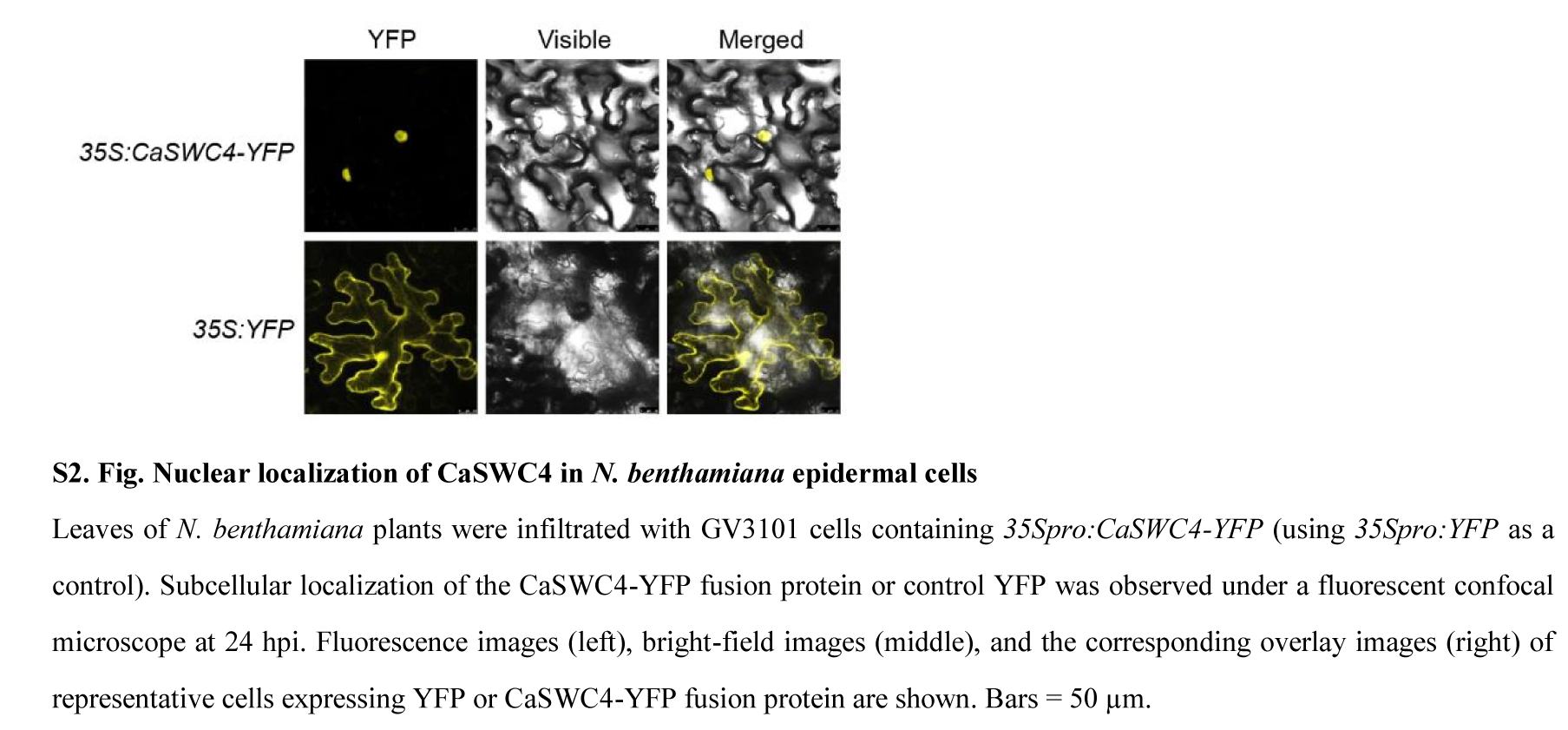

Supplement: S2 Fig — Leaves of N. benthamiana plants were infiltrated with GV3101 cells containing 35Spro:CaSWC4-YFP (using 35Spro:YFP as a control). Subcellular localization of the CaSWC4-YFP fusion protein or control YFP was observed under a fluorescent confocal microscope at 24 hpi. Fluorescence images (left), bright-field images (middle), and the corresponding overlay images (right) of representative cells expressing YFP or CaSWC4-YFP fusion protein are shown. Bars = 50 μm. (TIF) [file pgen.1010023.s005.tif]

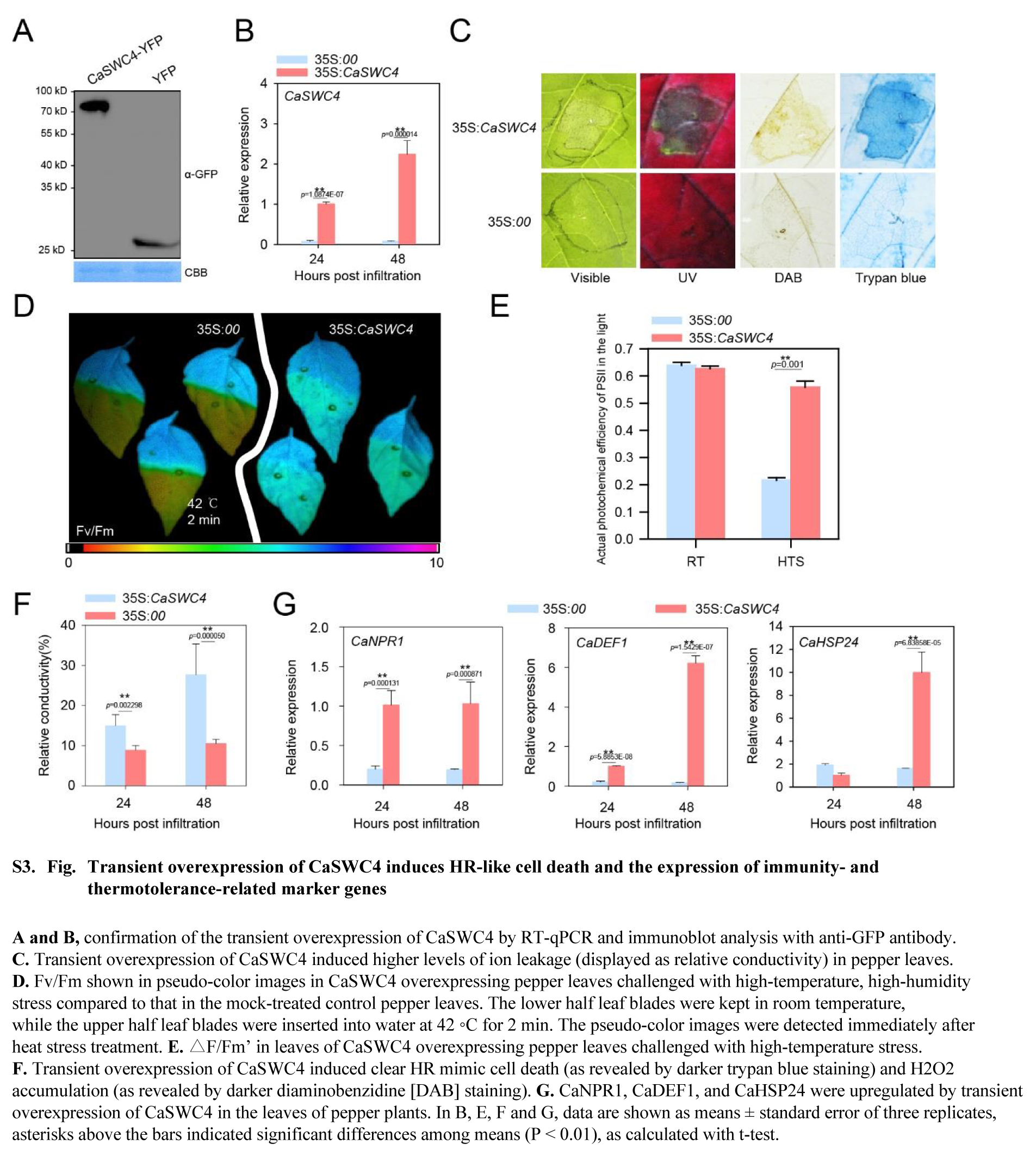

Supplement: S3 Fig — A and B, confirmation of the transient overexpression of CaSWC4 by RT-qPCR and immunoblot analysis with anti-GFP antibody. C. Transient overexpression of CaSWC4 induced higher levels of ion leakage (displayed as relative conductivity) in pepper leaves. D. Fv/Fm shown in pseudo-color images in CaSWC4 overexpressing pepper leaves challenged with HTS compared to that in the mock-treatment. The lower half leaf blades were kept in room temperature, while the upper half leaf blades were inserted into water at 42°C for 2 min. The pseudo-color image was detected immediately after HTS treatment. E. △F/Fm’ in leaves of CaSWC4 overexpressing pepper leaves challenged with HTS. F. Transient overexpression of CaSWC4 induced clear HR mimic cell death (as revealed by darker trypan blue staining) and H2O2 accumulation (as revealed by darker diaminobenzidine [DAB] staining). G. CaNPR1, CaDEF1, and CaHSP24 were upregulated by transient overexpression of CaSWC4 in the leaves of pepper plants. In B, E, F and G, data are shown as means ± standard error of three replicates, asterisks above the bars indicated significant differences among means (P < 0.01), as calculated with t-test. (TIF) [file pgen.1010023.s006.tif]

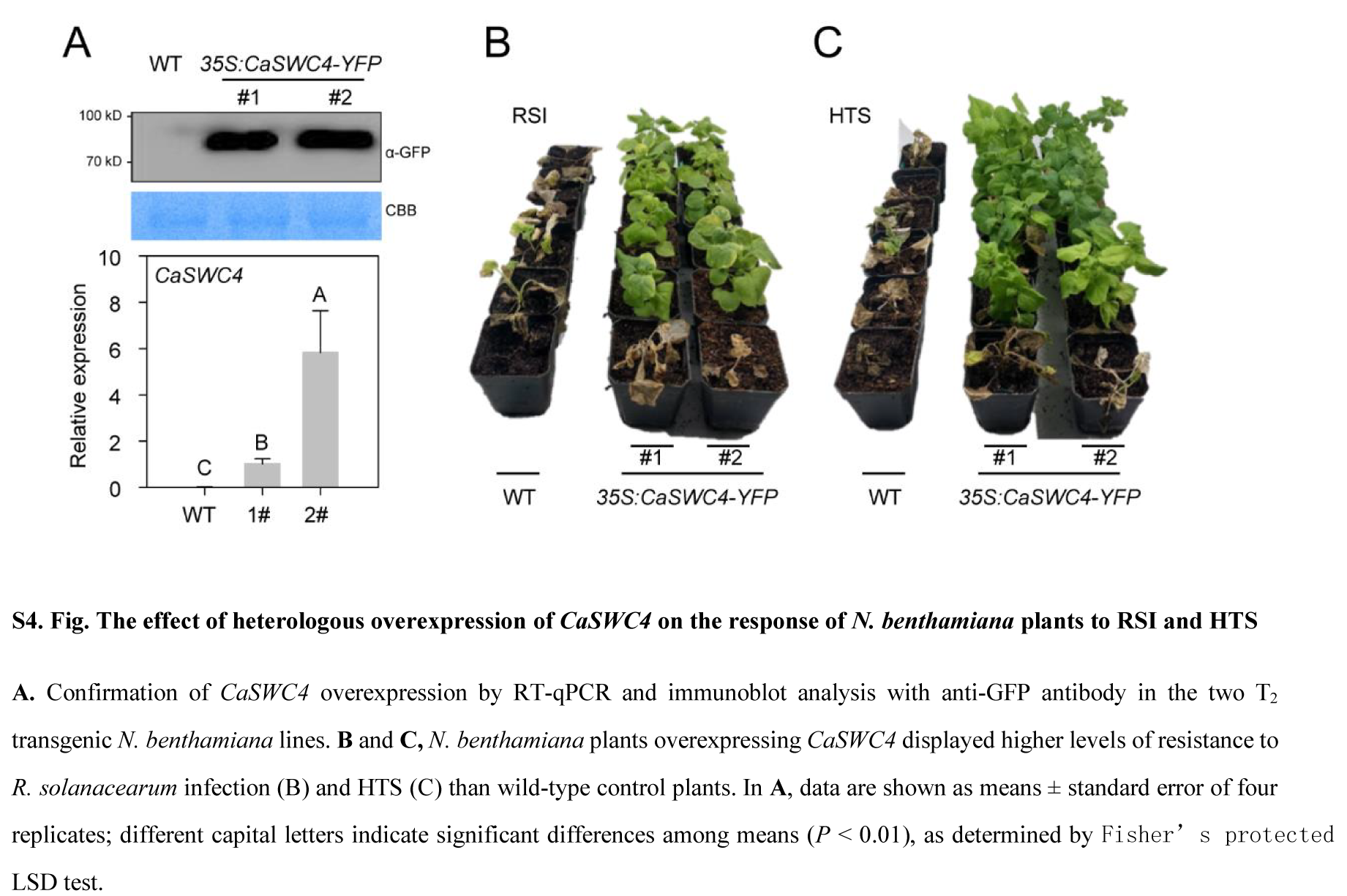

Supplement: S4 Fig — A. Confirmation of CaSWC4 overexpression by RT-qPCR and immunoblot analysis with anti-GFP antibody in the two T2 transgenic N. benthamiana lines. B and C, N. benthamiana plants overexpressing CaSWC4 displayed higher levels of resistance to R. solanacearum infection (B) and HTS (C) than wild-type control plants. In A, data are shown as means ± standard error of four replicates; different capital letters indicate significant differences among means (P < 0.01), as determined by Fisher’s protected LSD test. (TIF) [file pgen.1010023.s007.tif]

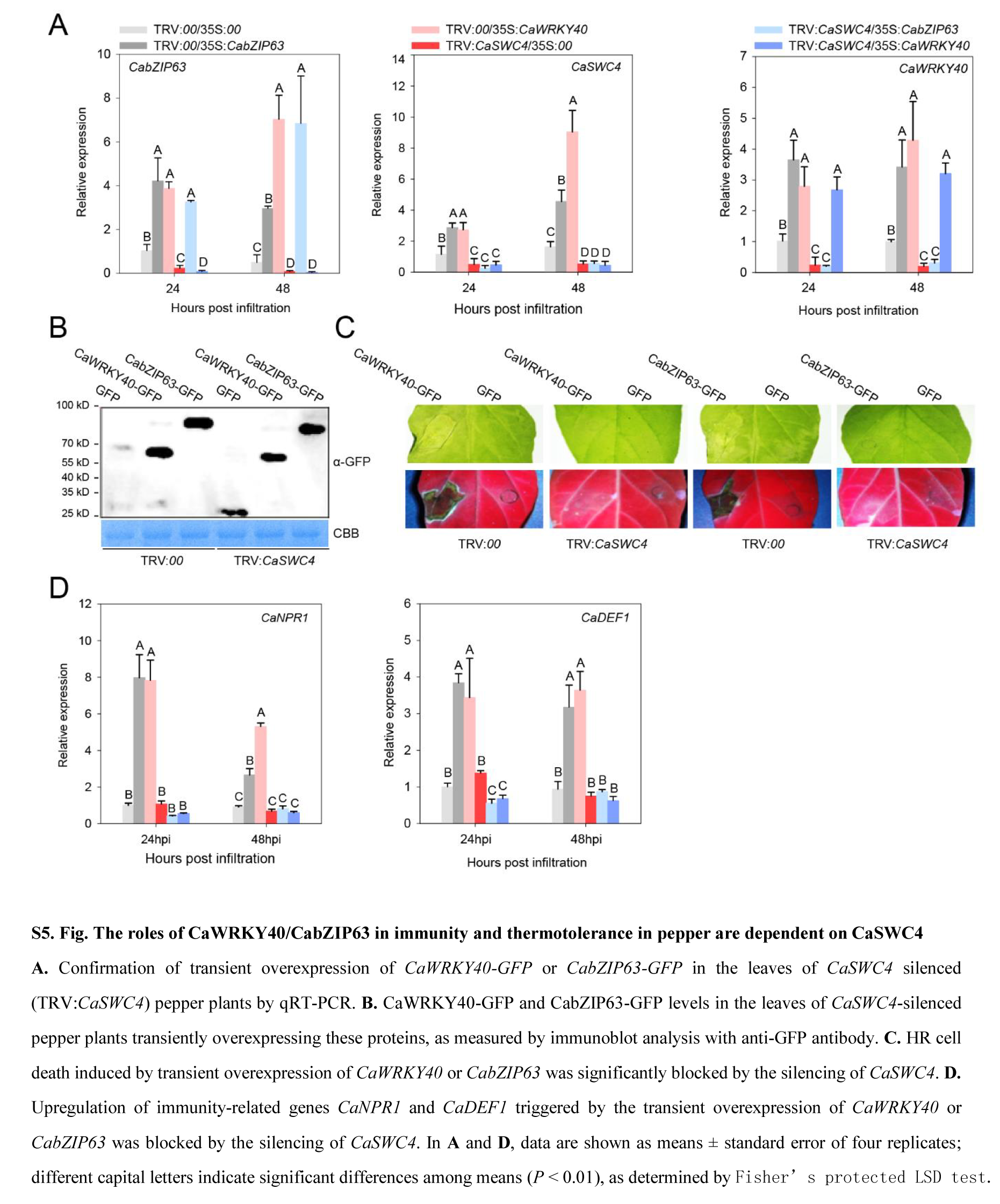

Supplement: S5 Fig — A. Confirmation of transient overexpression of CaWRKY40-GFP or CabZIP63-GFP in the leaves of CaSWC4 silenced (TRV:CaSWC4) pepper plants by qRT-PCR. B. CaWRKY40-GFP and CabZIP63-GFP levels in the leaves of CaSWC4-silenced pepper plants transiently overexpressing these proteins, as measured by immunoblot analysis with anti-GFP antibody. C. HR cell death induced by transient overexpression of CaWRKY40 or CabZIP63 was significantly blocked by the silencing of CaSWC4. D. Upregulation of immunity-related genes CaNPR1 and CaDEF1 triggered by the transient overexpression of CaWRKY40 or CabZIP63 was blocked by the silencing of CaSWC4. In A and D, data are shown as means ± standard error of four replicates; different capital letters indicate significant differences among means (P < 0.01), as determined by Fisher’s protected LSD test. (TIF) [file pgen.1010023.s008.tif]

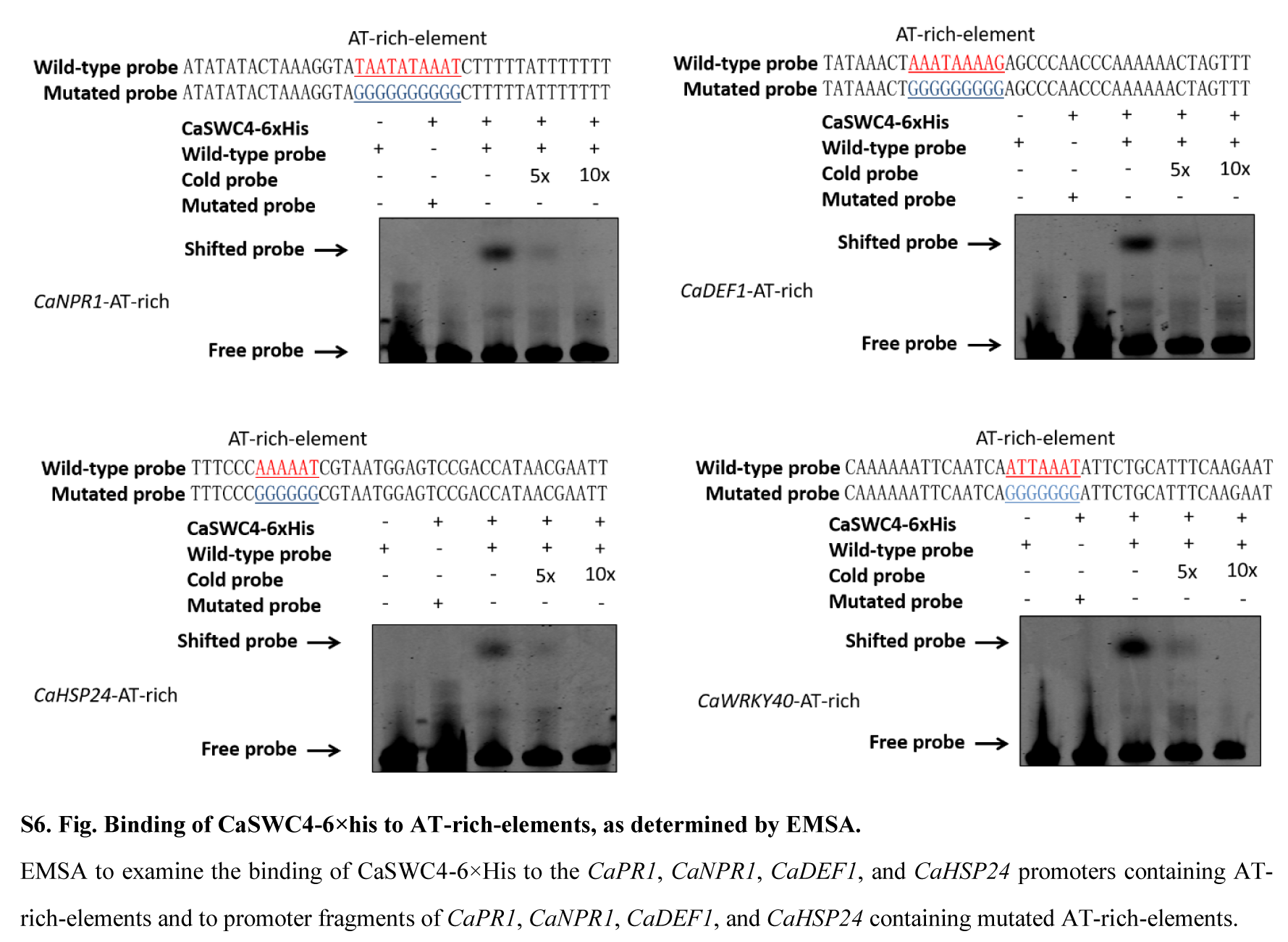

Supplement: S6 Fig — EMSA to examine the binding of CaSWC4-6×His to the CaPR1, CaNPR1, CaDEF1, and CaHSP24 promoters containing AT-rich-elements and to promoter fragments of CaPR1, CaNPR1, CaDEF1, and CaHSP24 containing mutated AT-rich-elements. (TIF) [file pgen.1010023.s009.tif]

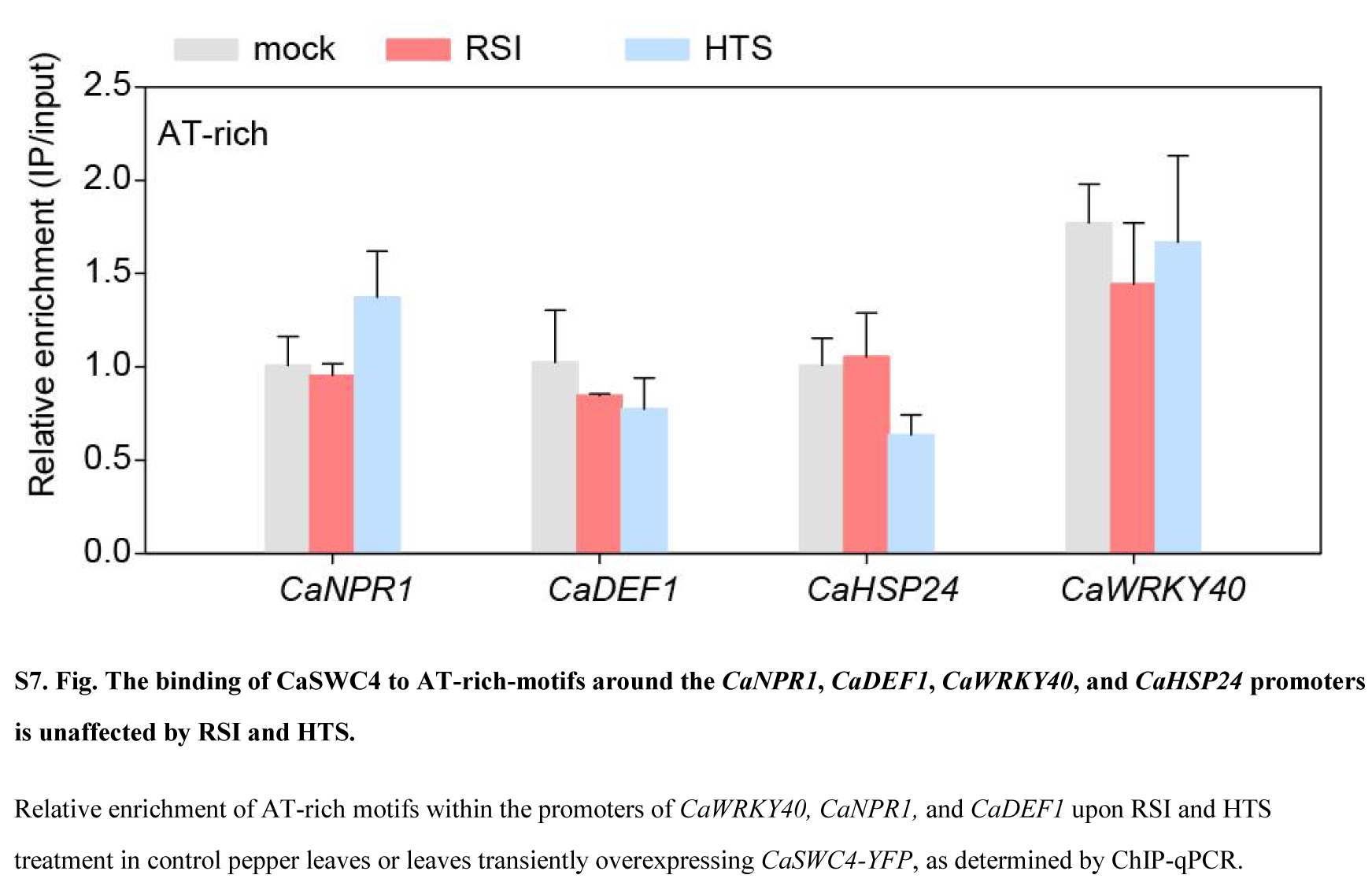

Supplement: S7 Fig — Relative enrichment of AT-rich motifs within the promoters of CaWRKY40, CaNPR1, and CaDEF1 upon RSI and HTS treatment in control pepper leaves or leaves transiently overexpressing CaSWC4-YFP, as determined by ChIP-qPCR. (TIF) [file pgen.1010023.s010.tif]

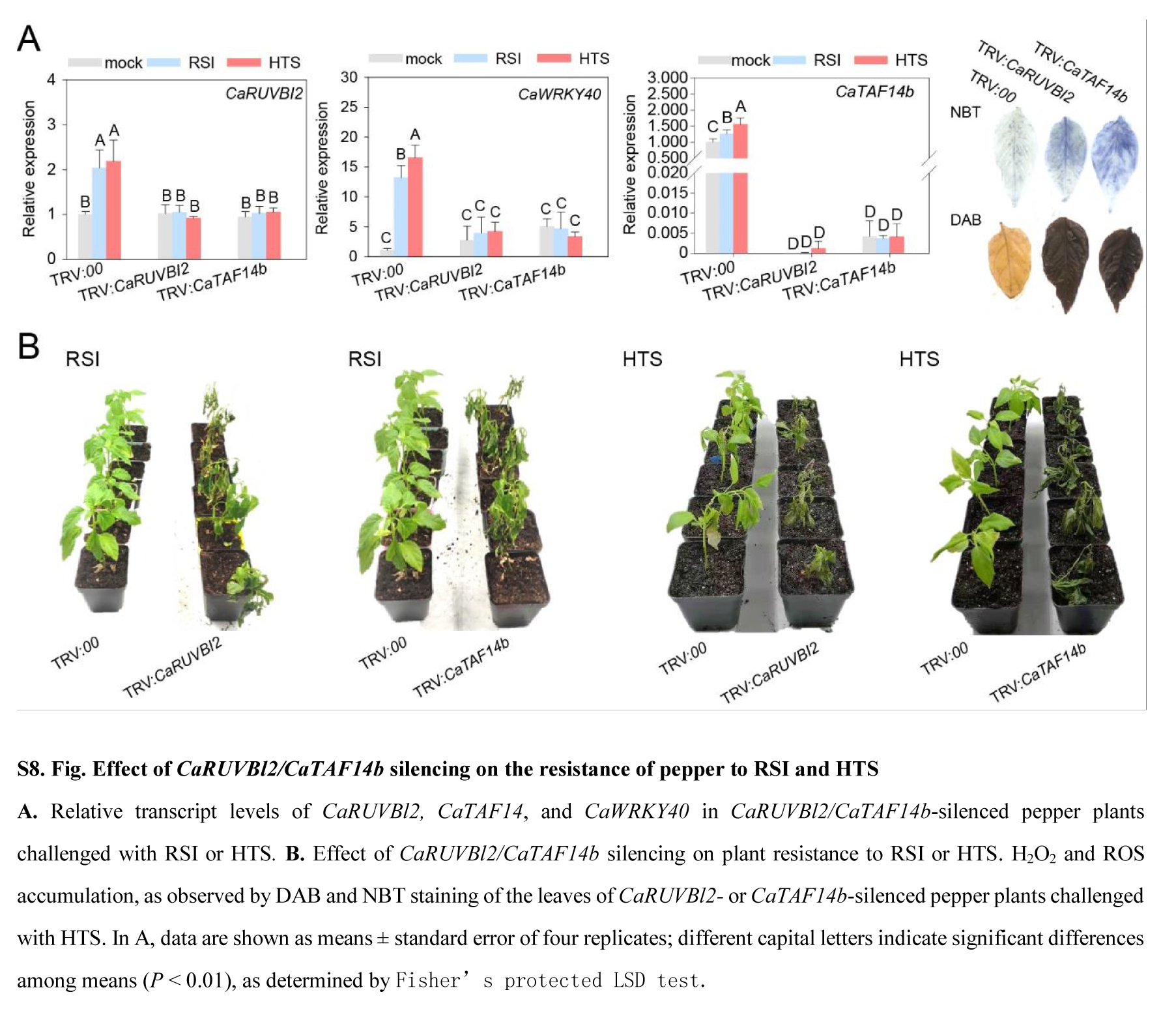

Supplement: S8 Fig — A. Relative transcript levels of CaRUVBL2, CaTAF14b, and CaWRKY40 in CaRUVBL2/CaTAF14b-silenced pepper plants challenged with RSI or HTS. B. Effect of CaRUVBL2/CaTAF14b silencing on plant resistance to RSI or HTS. H2O2 and ROS accumulation, as observed by DAB and NBT staining of the leaves of CaRUVBL2- or CaTAF14b-silenced pepper plants challenged with HTS. In A, data are shown as means ± standard error of four replicates; different capital letters indicate significant differences among means (P < 0.01), as determined by Fisher’s protected LSD test. (TIF) [file pgen.1010023.s011.tif]

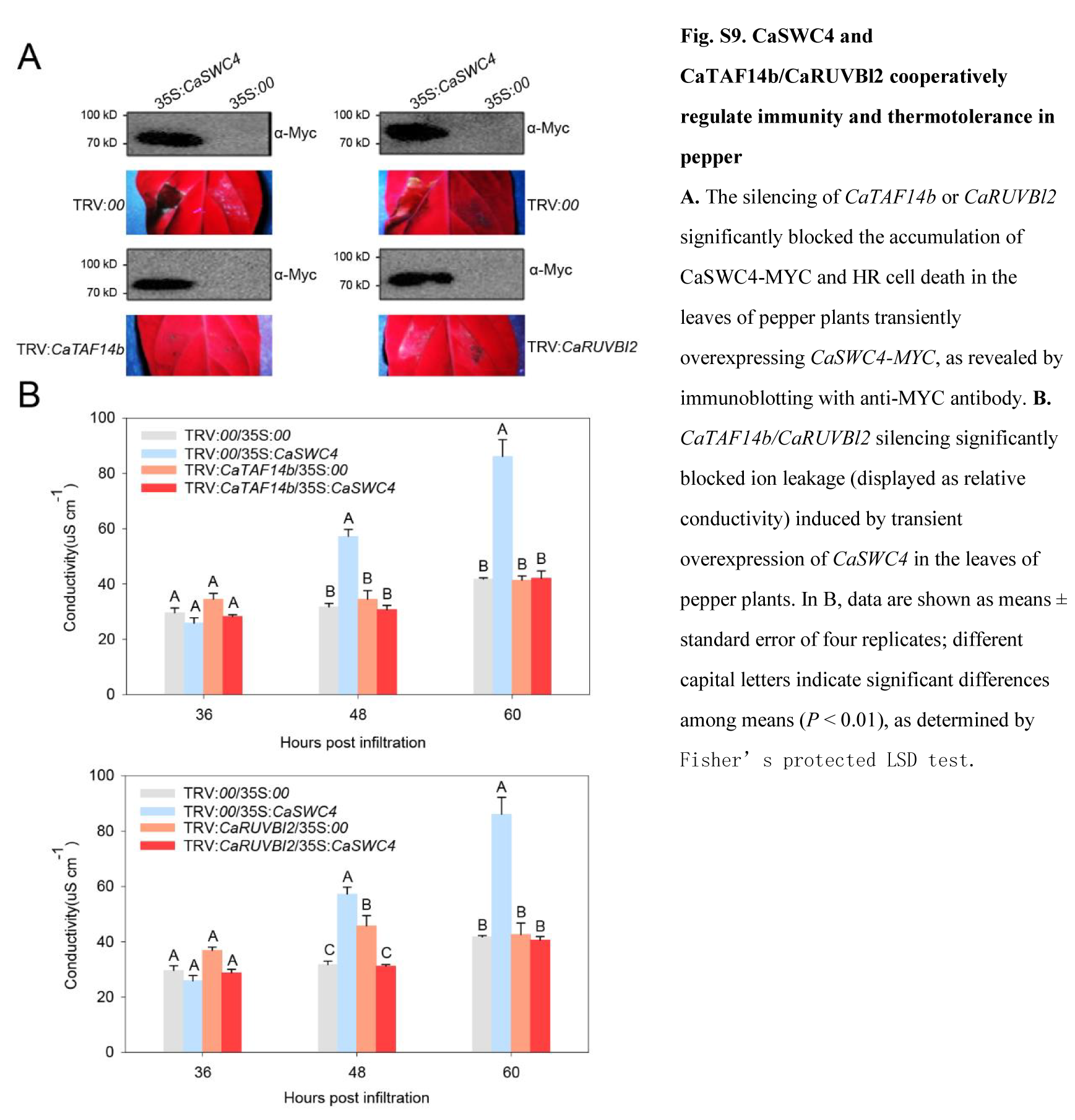

Supplement: S9 Fig — A. The silencing of CaTAF14b or CaRUVBL2 significantly blocked the accumulation of CaSWC4-MYC and HR cell death in the leaves of pepper plants transiently overexpressing CaSWC4-MYC, as revealed by immunoblotting with anti-MYC antibody. B. CaTAF14b/CaRUVBL2 silencing significantly blocked ion leakage (displayed as relative conductivity) induced by transient overexpression of CaSWC4 in the leaves of pepper plants. In B, data are shown as means ± standard error of four replicates; different capital letters indicate significant differences among means (P < 0.01), as determined by Fisher’s protected LSD test. (TIF) [file pgen.1010023.s012.tif]

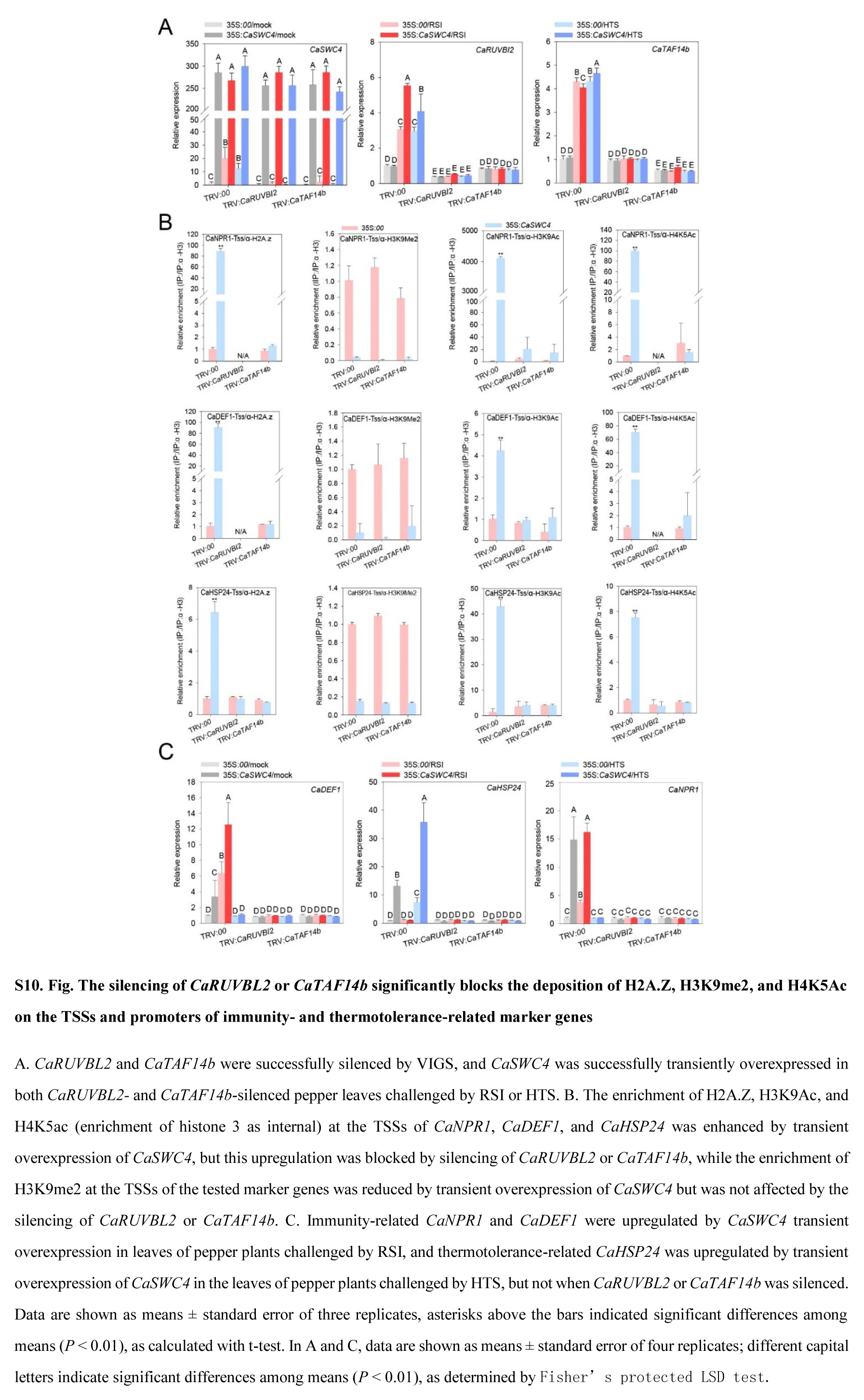

Supplement: S10 Fig — A. CaRUVBL2 and CaTAF14b were successfully silenced by VIGS, and CaSWC4 was successfully transiently overexpressed in both CaRUVBL2- and CaTAF14b-silenced pepper leaves challenged by RSI or HTS. B. The enrichment of H2A.Z, H3K9Ac, and H4K5ac (enrichment of histone 3 as internal) at the TSSs of CaNPR1, CaDEF1, and CaHSP24 was enhanced by transient overexpression of CaSWC4, but this upregulation was blocked by silencing of CaRUVBL2 or CaTAF14b, while the enrichment of H3K9me2 at the TSSs of the tested marker genes was reduced by transient overexpression of CaSWC4 but was not affected by the silencing of CaRUVBL2 or CaTAF14b. C. Immunity-related CaNPR1 and CaDEF1 were upregulated by CaSWC4 transient overexpression in leaves of pepper plants challenged by RSI, and thermotolerance-related CaHSP24 was upregulated by transient overexpression of CaSWC4 in the leaves of pepper plants challenged by HTS, but not when CaRUVBL2 or CaTAF14b was silenced. Data are shown as means ± standard error of three replicates, asterisks above the bars indicated significant differences among means (P < 0.01), as calculated with t-test. In A and C, data are shown as means ± standard error of four replicates; different capital letters indicate significant differences among means (P < 0.01), as determined by Fisher’s protected LSD test. (TIF) [file pgen.1010023.s013.tif]
